# Gene expression profiling in peanut using high density oligonucleotide microarrays

**DOI:** 10.1186/1471-2164-10-265

**Published:** 2009-06-12

**Authors:** Paxton Payton, Kameswara Rao Kottapalli, Diane Rowland, Wilson Faircloth, Baozhu Guo, Mark Burow, Naveen Puppala, Maria Gallo

**Affiliations:** 1 United States Department of Agriculture Cropping Systems Research Laboratory, Lubbock, Texas 79415, USA; 2 Texas Tech University, Department of Plant and Soil Science, Lubbock, Texas 79409, USA; 3 United States Department of Agriculture, National Peanut Research Laboratory, Dawson, Georgia, USA; 4 Crop Protection and Research Management Laboratory, Tifton, Georgia, 31793, USA; 5 Texas Agrilife Research, Lubbock, Texas 79403, USA; 6 New Mexico State University Agricultural Science Center, Clovis, New Mexico 88101, USA; 7 Institute of Food and Agricultural Sciences and the Genetics Institute, University of Florida, Gainesville, Florida 32611, USA

## Abstract

**Background:**

Transcriptome expression analysis in peanut to date has been limited to a relatively small set of genes and only recently has a significant number of ESTs been released into the public domain. Utilization of these ESTs for oligonucleotide microarrays provides a means to investigate large-scale transcript responses to a variety of developmental and environmental signals, ultimately improving our understanding of plant biology.

**Results:**

We have developed a high-density oligonucleotide microarray for peanut using 49,205 publicly available ESTs and tested the utility of this array for expression profiling in a variety of peanut tissues. To identify putatively tissue-specific genes and demonstrate the utility of this array for expression profiling in a variety of peanut tissues, we compared transcript levels in pod, peg, leaf, stem, and root tissues. Results from this experiment showed 108 putatively pod-specific/abundant genes, as well as transcripts whose expression was low or undetected in pod compared to peg, leaf, stem, or root. The transcripts significantly over-represented in pod include genes responsible for seed storage proteins and desiccation (e.g., late-embryogenesis abundant proteins, aquaporins, legumin B), oil production, and cellular defense. Additionally, almost half of the pod-abundant genes represent unknown genes allowing for the possibility of associating putative function to these previously uncharacterized genes.

**Conclusion:**

The peanut oligonucleotide array represents the majority of publicly available peanut ESTs and can be used as a tool for expression profiling studies in diverse tissues.

## Background

Cultivated peanut (*Arachis hypogaea *L.) is the second-most important legume in the world, with a total global production of 48 million tons [1]. Legumes are the second-most important food crop following grains, representing an important source of protein for humans and livestock in the North and South America, Africa, and Asia. Additionally, when considering oil production for cooking and fuels, peanut represents one of the highest value-added crops, with an annual worth of $1 billion to farmers and $6 billion to the overall economy in the U.S. alone.

Recent progress in functional genomics has enabled the study of plant responses at whole-transcriptome levels, revealing the complex nature of multi-genic responses in plants [2-4]. While genes and proteins expressed differentially under a variety of environmental perturbations and developmental stages have been identified in model plant systems such as *Arabidopsis *[2,5], studies on stress-induced or developmentally regulated genes in crop plants have been limited but are beginning to emerge [6-9]. While positional cloning and candidate gene approaches have begun to identify a number of structural genes or transcription factors controlling the larger response to abiotic and biotic stimuli [10,11], this work has been limited in peanut due to a lack of genomic data. Identification of such genes will have a significant effect on varietal development by traditional breeding and genetic engineering.

Greater attention is needed for genomic development in the Leguminosae. Despite its importance as both a cash crop and important staple, little is known about the genetic mechanisms in peanut that control disease resistance or susceptibility, stress tolerance, or pod development [12]. Although significant efforts have gone into legume genomics, there is a paucity of genomic data for peanut, bean, and chickpea compared to soybean, *Medicago truncatula*, and *Lotus japonicus *[4,8]. In peanut, marker technology is relatively young and only recently have genetic maps been published [13-15]. Although an initial cDNA microarray with 384 unigenes was published [16], there are no reports of high-density oligonucleotide microarray platforms in peanut. As part of our ongoing effort to identify the molecular mechanisms underlying peanut development and response to abiotic stress, we have designed a custom oligonucleotide microarray using all publicly available peanut ESTs. There are several advantages to the oligonucleotide microarray approach, including uniformity of hybridization, probe performance and specificity, and the flexibility of customization or probe addition as more sequences enter the public domain [17-20]. To test the utility of this array for expression studies in both vegetative and reproductive tissues and identify putatively pod-specific genes, we compared transcript abundance in pod, leaf, stem, root, and peg tissues. We present here, the utility of the first large-scale publicly available peanut microarray and establish the foundation for investigation of molecular responses on a transcriptome scale.

## Results and discussion

### Peanut microarray design

An oligonucleotide microarray containing 15,744 unique probes was created from 49,205 peanut ESTs available in Genebank (December 2007) as templates for probe design (Table 1). A total of 36,766 probes were designed using the server-based eArray platform from Agilent Technologies [21]. The remaining ESTs represented duplicates, sequences interspersed with long repeats, or a significant number of undetermined bases which failed to meet criteria required for accurate probe design. The initial set of 15,875 high quality probes with a cross hybridization potential of zero were used to query SWISPROT with BLASTx. The multiple matches from this query were saved and the best match that was better than E-10 was used to annotate each probe. Those probes not meeting the criterion for annotation were annotated as having unknown function. Probes annotated as "unknown" were binned into two categories: 1) probes not meeting the minimum criteria from the BLASTx query, and 2) probes matching a sequence (E-value < -10) annotated as unknown in SWISSPROT, i.e., "known unknowns". A final list of 14,352 probes was selected to create the probe group AH006 for microarray design (design id 017430) in addition to 536 Agilent controls and 856 random probes selected from the existing list of 14,352 probes.

**Figure 1:**
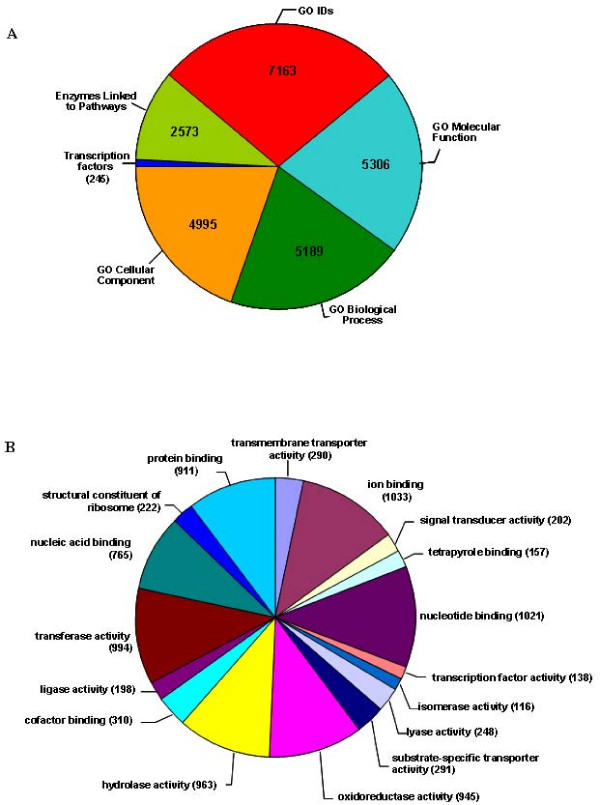
**Functional classification of unique, known genes on the AH006 peanut microarray**. A. Gene Ontology hits registered for the 5086 unique transcripts that could be assigned putative function based on Swiss-Prot query. B. Gene Ontology Molecular Functions for the AH006 array. Only known genes are shown to simplify the diagram.

Functional category enrichment based on Gene Ontology (GO) was performed for all 14,352 probes present in the array using the Blast2GO search tool [22]. Query against SWISPROT resulted in the annotation of 5,086 known genes and 6,793 transcript probes with unknown function. Figure 1A shows GO functional groups for known transcripts represented on the AH006 array and a detailed description of the GO molecular function (MF) cluster is displayed in Figure 1B which indicates uniform distribution of probes with binding and catalytic functions. This represents ~24% of genic content, given that the total number of genes in peanut is estimated to be 50,000 [23]. The peanut ESTs used in this design were from libraries representing diverse tissues, although root, stem, and cotyledon are under-represented (Table 1). Therefore, these microarray probes have a broad utility for tissue specific transcripts expressed under a variety of conditions. Furthermore, the use of the Agilent system allows for flexibility in future array versions as additional ESTs can be added from the public domain.

**Table 1:** Source tissue and number of ESTs from each library used to design the AH006 peanut microarray.

**Tissue**	**Treatment**	**ESTs for Array Design**
Leaf	control	13884
	drought + Aspergillus	2046
Seed/Pod	control	10242
	drought + Aspergillus	14328
Root	control	6123
Cotyledon	control	2533
Stem	control	49
**Total**		**49205**

A full description of the array is available in the Gene Expression Omnibus database as platform GPL6661.

### Microarray quality

**Figure 2:**
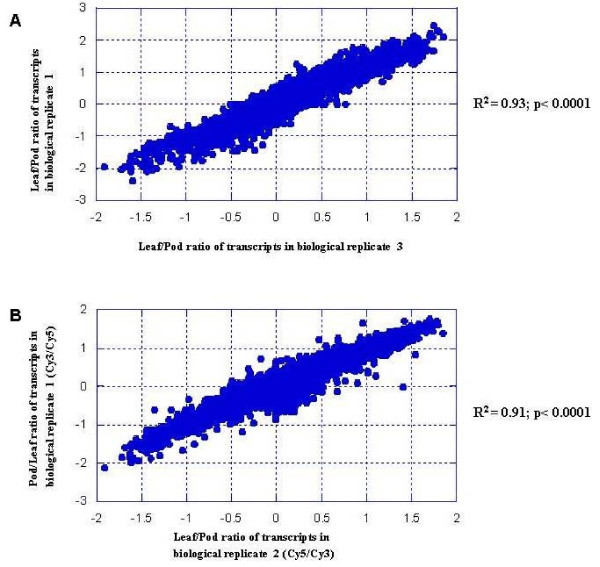
**The correlation of normalized ratios between biological replicates and dye swaps**. A. The correlation of normalized ratios between leaf vs pod from two biological replicates R^2 ^= 0.93. B. The correlation of normalized ratios between the same leaf vs pod with dye swapped in two different biological replicates, R^2 ^= 0.91.

The quality of the microarray was evaluated using the two comparisons: (1) two biological replicates of the same tissue-type labeled with the same dye and (2) the same tissue from two biological replicates labeled with either Cy3 or Cy5 dyes. The correlation coefficients of log transformed normalized ratios between the two replicates and two different dyes (dye-swap) were calculated by KaleidaGraph 3.6 (Synergy Software, USA) (Figures 2A and 2B). For pod and leaf comparisons, the correlation coefficient between the biological replicates labeled with Cy3/Cy5 was 0.93 (Figure 2A). Reciprocal hybridizations (dye-swaps) were utilized for all tissue comparisons to avoid dye bias. The correlation coefficient for the same pod and leaf tissues in a dye-swap experiment where the same tissue was labeled with Cy5 in one biological replicate and Cy3 in another biological replicate was 0.91 (Figure 2B). For labeling, 50 ng to 1 μg total RNA is needed for Agilent custom arrays unlike cDNA arrays which require 20 μg to 30 μg. This is very important for profiling samples containing limited amounts of RNA or small structures such as floral parts and developing pods. Further the 8 × 15 k array design has the feature of eight independent arrays in one slide, which is not only cost effective but also can reduce variation among the arrays within a slide.

### Tissue specific gene expression using the peanut oligonucleotide microarray

**Figure 3:**
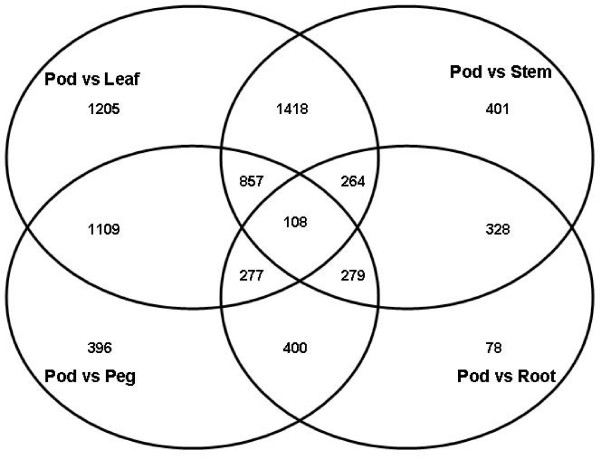
**Venn diagram showing number of differentially expressed tissue specific transcripts**.

**Figure 4:**
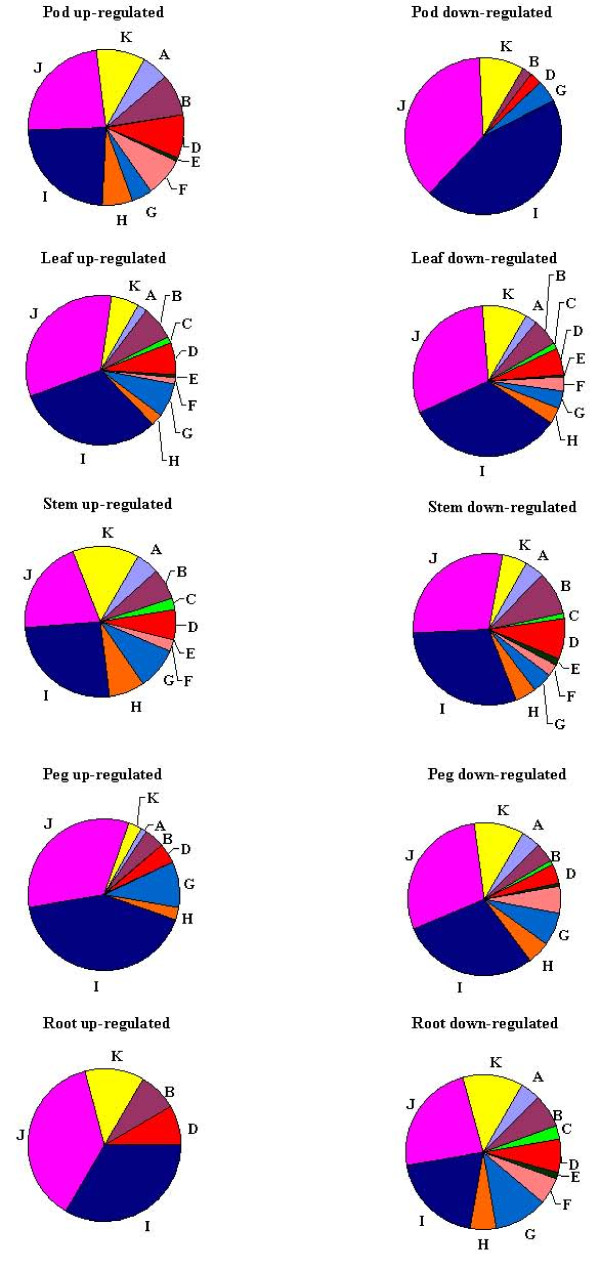
**Gene Ontology terms for biological process classification for genes showing tissue-specific expression patterns in pod, leaf, stem, peg and root abundant transcripts (also described in Table 1)**. A. multi-cellular organismal process; B. localization; C. multi-organism process; D. establishment of localization; E. growth; F. reproductive process; G. biological regulation; H. developmental process; I. metabolic process; J. cellular process; K. response to stimuli.

In recent studies, leaf, root, seed coat, and cotyledon tissues were utilized for global expression profiling in soybean [6,8] and leaf and bud tissues were used to test a spotted cotton oligonucleotide microarray for tissue-specific gene expression analysis [24]. Four pairs of tissue comparisons were performed for each tissue in the present study. These comparisons resulted in the list of statistically-significant, differentially expressed genes in each tissue shown in Figure 3, Additional file 1. The entire data set can be accessed at the Gene Expression Omnibus (GEO) database as platform GPL6661 and series GSE11365. Additionally, GO annotations based on biological process (BP) are presented in Figure 4 for those transcripts showing tissue-specific expression patterns. Transcripts showing at least two-fold difference in abundance (expression ratio ≥ 2 or ≤ 0.5) at a P-value ≤ 0.05 were classified as differentially expressed and those with differential expression unique to a single tissue were considered as having putatively tissue-specific functions [25]. In summary, there were 4046 transcripts representing 3650 gene functions that were differentially expressed in at least one tissue comparison. Of these transcripts, 1204, 401, 78, and 396 showed tissue-specific differential expression patterns in leaf, stem, root, and peg, respectively (Figure 3). Two-hundred-eleven gene transcripts were differentially expressed in all four comparisons, 161 pod-abundant and 50 that were significantly more abundant in leaf, stem, root, and peg compared to pod.

Gene expression profiles of different tissues provide information about the biological function of the genes expressed in those tissues [24,26]. For the pod abundant pool, only 21 transcripts could be assigned a putative function based on BLAST analysis. All tissues showed similar GO BP enrichments associated with metabolic processes (I), cellular processes (J), and response to stimuli (K). While peanut pod undisputedly is the most important organ from an agronomic perspective and the genes specifically up-regulated in that tissue are of interest, other tissue-specific genes or expression patterns may reveal significant information related to productivity, disease resistance, development, and physiological response. Figure 4 shows that the functional roles of putative tissue-specific genes are similar for leaf, stem, and peg compared to root. While this is not surprising given the similarities of genes highly expressed in green leaves or stems, it should be noted that the majority of peanut EST sequences in the public domain are from leaf and pod. However, despite the absence of a large number of ESTs from root libraries, there are genes whose expression appears to be root specific.

### Genes and pathways identified in pods

Due to paucity of information on peanuts in global repositories like NCBI, only half of the pod-abundant transcripts could be meaningfully annotated (Additional file 2). Two major categories of transcripts, namely storage proteins and desiccation-related proteins, were identified in pods. Five transcripts related to seed storage proteins such as globulin, conglutin and glycinin were abundant in pod tissues. The desiccation-related transcripts over-represented included seed maturation protein, LEA, early methionine labeled (EM), legumin, plasma membrane intrinsic proteins (aquaporins) and desiccation related pcc13-62 proteins. In most higher plants the later seed maturation phase is characterized by a desiccation phase during which number of proteins distinct from the storage proteins are accumulated in embryos. According to their accumulation pattern it has been suggested that these particular proteins, called Late Embryogenesis Abundant (LEA) could be involved in seed desiccation tolerance [27,28]. In addition to their expression during seed desiccation, many of the genes coding for LEAs can be highly induced in immature seeds or activated in vegetative tissues upon osmotic stress [29], indicating that they are, in part, regulated at the transcriptional level [30]. On the other hand EM proteins could be responsible for the maintenance of a minimal water content allowing preservation of cell content in dried seeds [30,31].

Utilizing the blast2GO tool, twelve transcripts with an Enzyme Commission (EC) number were mapped to twenty five different Kyoto Encyclopedia of Genes and Genomes (KEGG) pathways. Of these, 18 pathways relevant to pods were presented in Additional File 3. As expected, five major pathways leading to the production of sugars and starch involving the enzymes UDP-glucose pyrophosphorylase (EC:2.7.7.9) and dTDP-glucose 4-6-dehydratase (EC:4.2.1.46) were identified. Peanut being an oilseed crop, the pathways leading to lipid metabolism (2 pathways) and sulfur containing amino acid metabolism (5 pathways) were predominant in pods. Peroxidase enzyme (EC:1.11.1.7) found abundant in pods have multiple roles in plants. Apart from its reactive oxygen scavenging and water-stress signaling activity [32], the peroxidase enzyme also catalyses phenylpropanoid biosynthesis and phenylalanine metabolism resulting in defense compounds which may protect the developing peanut pods in the soil. Two enzymes involved in pyruvate metabolism phosphoenolpyruvate carboxylase (EC:4.1.1.31) and hydroxyacylglutathione hydrolase (EC.3.1.2.6) were also found to be more abundant in pods. Pyruvate thus generated may be involved in biosynthesis of secondary metabolites like terpenoids by the action of 1-deoxy-d-xylulose 5 phosphate synthase (EC:2.2.1.7). Together the pathway analyses suggests that in pod tissues apart form basic starch and lipid metabolism, secondary metabolites such as phenylpropanoids and terpenoids are also synthesized and may impart defense for developing pod tissues in soil.

### Validation of array data with quantitative real-time PCR

Quantitative real time PCR has become a gold standard for the gene expression and generally used for validation of microarray results [33]. To validate the microarray data from our study, quantitative real time PCR (qRT-PCR) analyses were performed on the same mRNA samples used for the microarray experiments. Eight differentially-expressed transcripts, 7 pod-enriched and 1 pod-deficient, were selected for qRT-PCR analysis (Table 2). The relative expression pattern of all eight selected genes resembled respective microarray expression patterns (Table 3) and suggested that microarray analyses utilizing the current array were highly reliable and accurate.

**Table 2:** List of primers for qRT-PCR analysis of tissue-abundant genes.

**Gene name**	**Accession #**	**Primer name**	**Primer sequence (5'-3')**	**Primer effeciency**	**Amplicon size (bp)**
Glycinin precursor	gi| 146771807	GLY F-	TATGATGATGACGATCGACGACCACG	1.738	82
		GLY R-	TGCATAGTGTTTCCTCCACTCCGT		
Late embryogenesis abundant protein 2	gi| 110810624	LEA2 F	TAGTTCGGGTTGTAGTAGCAGGGT	1.831	99
		LEA2 R	AAGGTTCCATCTTCTCGCCGATGT		
Protein disulfide-isomerase precursor	gi| 56690261	PDI F	AGGGTTCCGATCTGCTTCCTCTTT	1.823	96
		PDI R	AAACTCCTTCTCTTTGGCCTCCGA		
Putative GPI anchored protein	gi| 110811592	GPI-AP F	AAATAGAGGACGAGCCATGCGAGA	1.603	89
		GPI-AP R	AGGTTTGGAATGTTTGCGCTGGAG		
Plasmamembrane intrinsic protein 2	gi| 149221199	PIP2 F	AAGACAAGCCCTGGGATGACCATT	1.832	96
		PIP2 R	CTGCCCTCAAGATGAATTGGTGGT		
Transmembrane emp24 domain-containing protein 2 precursor	gi| 149222425	emp24 F	ACACGAATGAGAGCACACGAAAGC	1.812	88
		emp24 R	ATGACCTGCAGTGCACTAACACCA		
Dessication-related protein PCC13-62	gi| 110811067	DRP F	TGGAGAATCTCTACATCCCTCCT	1.854	99
		DRP R	TCCCAGTGAGGCCAACATAAGGAA		
Lipoxygenase 4	gi| 126159580	LOX4 F	TATTCAAGGGAGGGTGGTCTCACT	1.796	90
		LOX4 R	AGGGATCCTGGCAAACAGGGAAAT		

**Table 3:** Expression pattern of peanut pod abundant transcripts.

		**Microarray fold change**				**Quantitative real-time PCR fold change**			
**Probe**	**Gene name**	**Leaf**	**Stem**	**Peg**	**Pod**	**Leaf**	**Stem**	**Peg**	**Pod**

AH39002	desiccation-related protein **(DRP)**	38	61	46	27	2467	69811	2126	23567
AH46647	lipoxygenase 4 **(LOX4A)**	0.16	0.33	0.29	0.27	0.25	0.62	0.3	0.2
AH35494	transmembrane emp24 domain-containing protein **(emp24)**	4	3	3	3	6	6	2	4
AH27472	protein disulfide-isomerase precursor **(PDI)**	9	4	3	4	51	20	9	9
AH40559	late embryogenesis abundant protein 2 **(LEA2)**	29	43	38	21	268	983	113	345
AH19851	glycinin precursor **(GLY)**	95	106	103	29	228	612	109	324
AH32426	aquaporin PIP2-1 **(PIP2)**	3	4	3	6	5	7	8	17
AH30432	putative GPI anchored protein **(GPI-AP)**	9	9	8	6	47	1655	24	580

Microarray and quantitative real time PCR expression values are mentioned as fold change of transcript in pods compared to different tissues.

## Conclusion

Peanut, being an under represented crop in terms of genome sequencing and physical mapping, needs a comprehensive tool for dissecting complex mechanisms of development and tolerance to biotic and abiotic stresses. To attain this broad objective, we have designed and characterized a high density oligonucleotide microarray suitable for transcript profiling of various peanut tissues. Analysis of pod abundant transcripts suggested the presence of distinct pathways involved in generation of secondary metabolites apart from the accumulation of transcripts for storage and desiccation-related protein. These peanut microarrays are publicly available and can be upgraded with additional oligonucleotides designed from subsequent sequencing efforts from the peanut research community. The expression profiles generated by these peanut microarrays will provide starting points for in-depth studies on candidate genes that can be utilized in reverse genetics to assign gene functions.

## Methods

### Plant tissue

Field grown plants of peanut cultivar FlavRunner 458 were used for tissue collection. The harvested tissue from leaves, pegs, stem, root and pods were immediately frozen in liquid nitrogen and stored at -80°C until further analysis.

### RNA extraction

Total RNA from different tissue was isolated using the RNeasy Plant Minikit (Qiagen, Valencia, CA). Pooled frozen tissue from five plants were ground to a fine powder in liquid nitrogen and approximately 100 mg of homogenized tissue was used for total RNA isolation according to manufacturer's protocol, except the homogenized seed tissue was initially extracted in 600 μl of RLT buffer and during purification, samples were incubated in buffer RW1 for 5 min during the column washing step. RNA samples were treated with Turbo DNAfree (Ambion, Inc., Austin, TX) prior to cDNA synthesis.

### cRNA synthesis

An aliquot of 450 ng of total RNA was used for cDNA synthesis utilizing the Low RNA Input Fluorescence Linear Amplification Kit (Agilent Technologies). Resulting cDNA was transcribed into cRNA and labeled with either cyanine 3 or cyanine 5-labeled nucleotides (Perkin Elmer, Wellesley, MA) using T7 RNA polymerase (Agilent Technologies). Labeled cRNA was purified with RNeasy Mini columns (Qiagen, Valencia, CA). The cRNA quality and quantity were determined spectrophotometrically using a NanoDrop ND-1000 spectrophotometer.

### Oligonucleotide microarray hybridization

**Figure 5:**
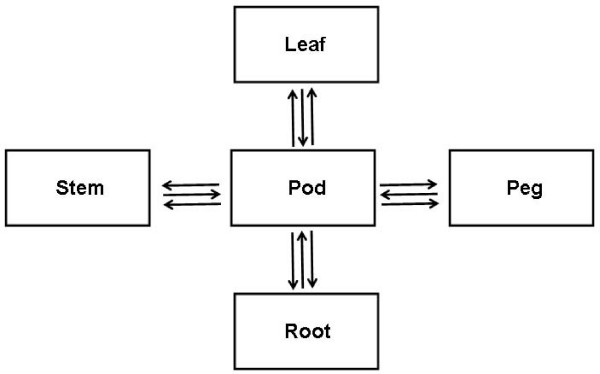
**Diagrammatic representation of microarray experimental design**. The arrow represents Cy5 and the end of the arrow represents Cy3.

Labeled cRNA from pod tissue was hybridized in combination with different tissues (Figure 5) using the *in situ *hybridization kit from Agilent Technologies. A total of 5 tissue samples were compared in three biological replicates with dyes swapped in the second biological replicate. Arrays were incubated at 65°C for 17 h in rotating hybridization chamber. Arrays were washed at room temperature under constant agitation for 10 minutes in 6× SSC with 0.005% Triton X-102 followed by a 5 minutes in cold, 0.1× SSC, 0.005% Triton X-102.

### Image scanning and data analysis

Arrays were scanned using a GenePix^® ^4000B microarray scanner at 5-μm resolution and images were saved as uncompressed tagged image files. For detection of significant differentially expressed genes, each slide image was processed by Agilent Feature Extraction software (version 9.1). This software measured Cy3 and Cy5 signal intensities of whole probes. Since dye bias tends to be signal intensity-dependent, probe sets for dye normalization were selected by rank consistency. Normalization was done by locally weighted linear regression (LOWESS). Ratios were log-transformed and significance values (P-value) were calculated based on a propagate error model and universal error model. In this analysis, the threshold of significant differentially expressed genes was determined with a p-value ≤ 0.05 (p-value is a measure of the confidence that the feature is not differentially expressed). Low-quality spot data generated due to artifacts were eliminated prior to data analysis. Processed intensities from feature extraction analysis were imported into the TIGR Multiexperiment Viewer software (MEV 4.1) and significant genes at a p-value of ≤ 0.05 and more than two-fold difference in expression were defined as differentially expressed.

### Annotation

The Gene Ontology functional annotation tool Blast2GO [22] was utilized to assign GO ids, enzyme commission numbers, and mapping to Kyoto Encyclopedia of Genes and Genomes (KEGG) pathways. The Blast2GO tool also enabled statistical analysis related to over representation of functional categories based on a Fisher Exact statistic methodology.

### Gene expression analysis using real time-PCR

#### cDNA synthesis and Primer Design

Total RNA samples were treated with Turbo DNAfree (Ambion, Inc., Austin, TX) prior to cDNA synthesis. One microgram of total RNA was used to synthesize first strand cDNA using SuperScript First Strand Synthesis system for RT-PCR (Invitrogren, CA). The primers for pod abundant genes and actin standard were designed using Integrated DNA Technologies primer designing tools. The efficiency of the primer pairs was determined on cDNA derived from the pod of FlavRunner 458 cultivar using a 1:2 serial dilution series. Primer efficiency reactions were performed in triplicate in volumes of 25 μL using SuperArray SYBRGreen reaction mix (SuperArray Bioscience Corp., MD). Reactions were subjected to real-time qRT-PCR using the Roche LightCycler 480 Real-Time PCR System and data analyzed using the LightCycler 480 quantification software (Roche Biochemicals, Indianapolis, IN) [12].

#### Real-Time qRT-PCR Conditions

Samples were analyzed in a 25 μL volume using the Roche LightCycler 480 (Roche Biochemicals, Indianapolis, IN). Reactions were performed in triplicate using cDNA templates from five tissues samples for each gene. A master mix of SYBRGreen and primers was prepared for each primer pair. RT-PCR reactions were performed on 40 ng total RNA with 400 nM specific primers under the following conditions: one cycle of denaturation at 95°C for 10 min followed by 40 cycles of 95°C for 15 sec (denaturation) and 60°C for 15 sec (annealing and elongation). The PCR reaction was followed by a melting curve program (60 – 95°C with a heating rate of 0.1°C per second and a continuous fluorescence measurement) and then a cooling program at 40°C. Negative controls lacking reverse transcriptase were run with all reactions. PCR products were also run on agarose gels to confirm the formation of a single product at the desired size. Crossing points for each transcript were determined using the 2^nd ^derivative maximum analysis with the arithmetic baseline adjustment. Crossing point values for each gene were normalized to the respective crossing point values for the reference gene actin. Data are presented as normalized ratios of genes along with error standard deviations estimated using the Roche Applied Science E-method [34].

## Authors' contributions

PP was responsible for the conception and design of the experiment and final revisions of the manuscript. PP and KRK designed the array and performed all data analysis and interpretation. KRK carried out the tissue collection, performed RNA extractions, array hybridizations, and real-time PCR. DR and WF assisted in tissue collection and participated in data interpretation and preparation of the manuscript. BG generated cDNA libraries and contributed to array design and preparation of the manuscript. MB and NP provided seed and contributed to data analysis and manuscript preparation. MG contributed to the conception of the experiment and manuscript preparation. All authors have read and approved the final manuscript.

## Supplementary Material

Additional file 1 **Differentially expressed tissue specific genes**. This table includes the list of statistically significant (p < 0.05) differentially expressed genes, including fold changes and functional descriptions, for leaf, stem, peg, and root tissue-specific genes. Click here for file

Additional file 2 **Pod abundant transcripts compared to all other tissues**. The data provided represent the list of differentially expressed pod abundant transcripts. GO mapping and annotation of probe sequences was performed by Blast2go tool (version 2.2.3). Click here for file

Additional file 3 **Pathways catalyzed by pod specific enzymes**. This figure includes eighteen different pathways catalyzed by 12 pod specific enzymes. Click here for file
