# Supplementary material for: Gene expression profiling in peanut using high density oligonucleotide microarrays
Source: BMC Genomics. 2009 Jun 12;10:265. doi: 10.1186/1471-2164-10-265 (PMC2703657; doi:10.1186/1471-2164-10-265)
Supplement: Additional file 3 — Pathways catalyzed by pod specific enzymes. This figure includes eighteen different pathways catalyzed by 12 pod specific enzymes. [file 1471-2164-10-265-S3.pdf]

Additional File 2

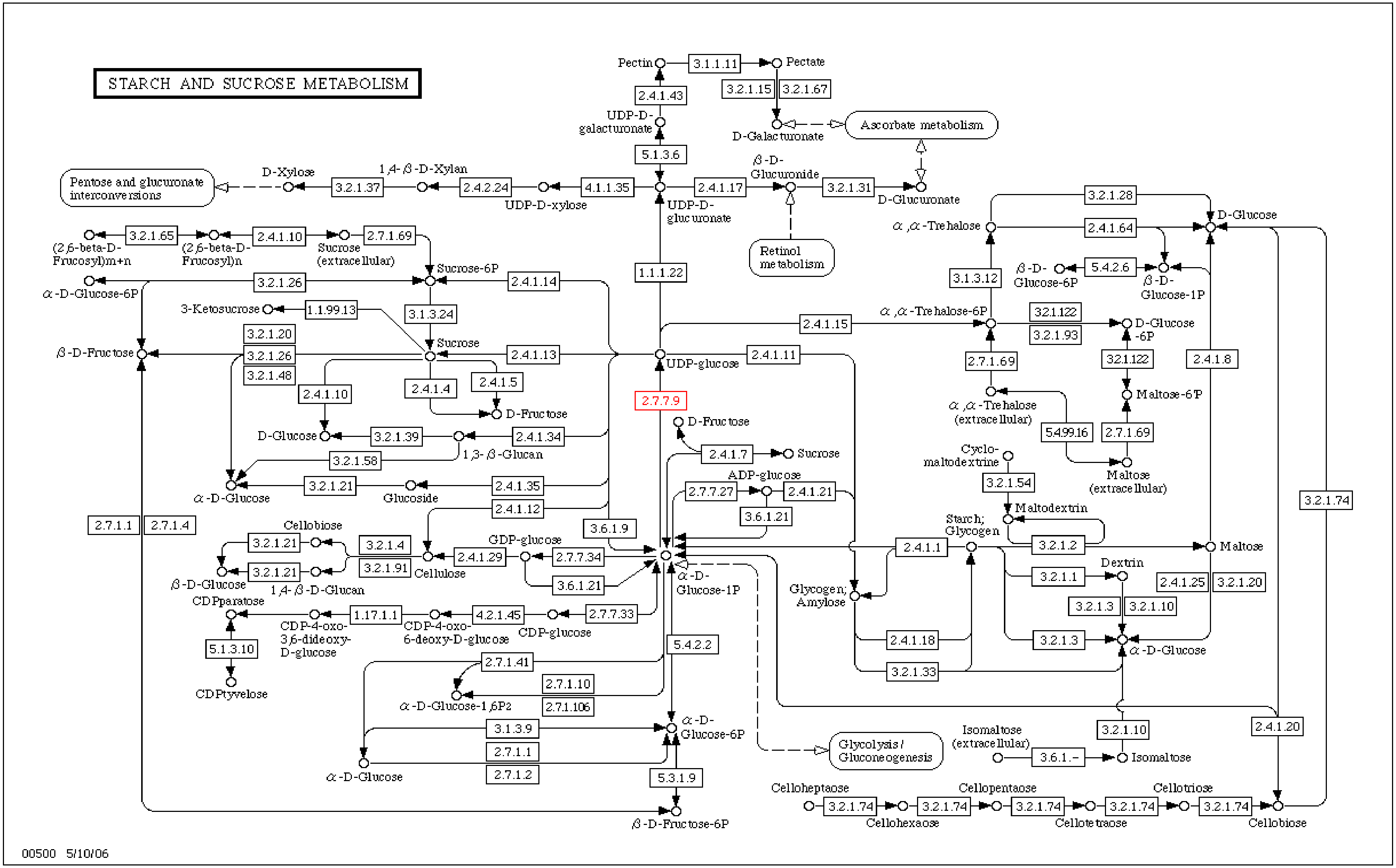

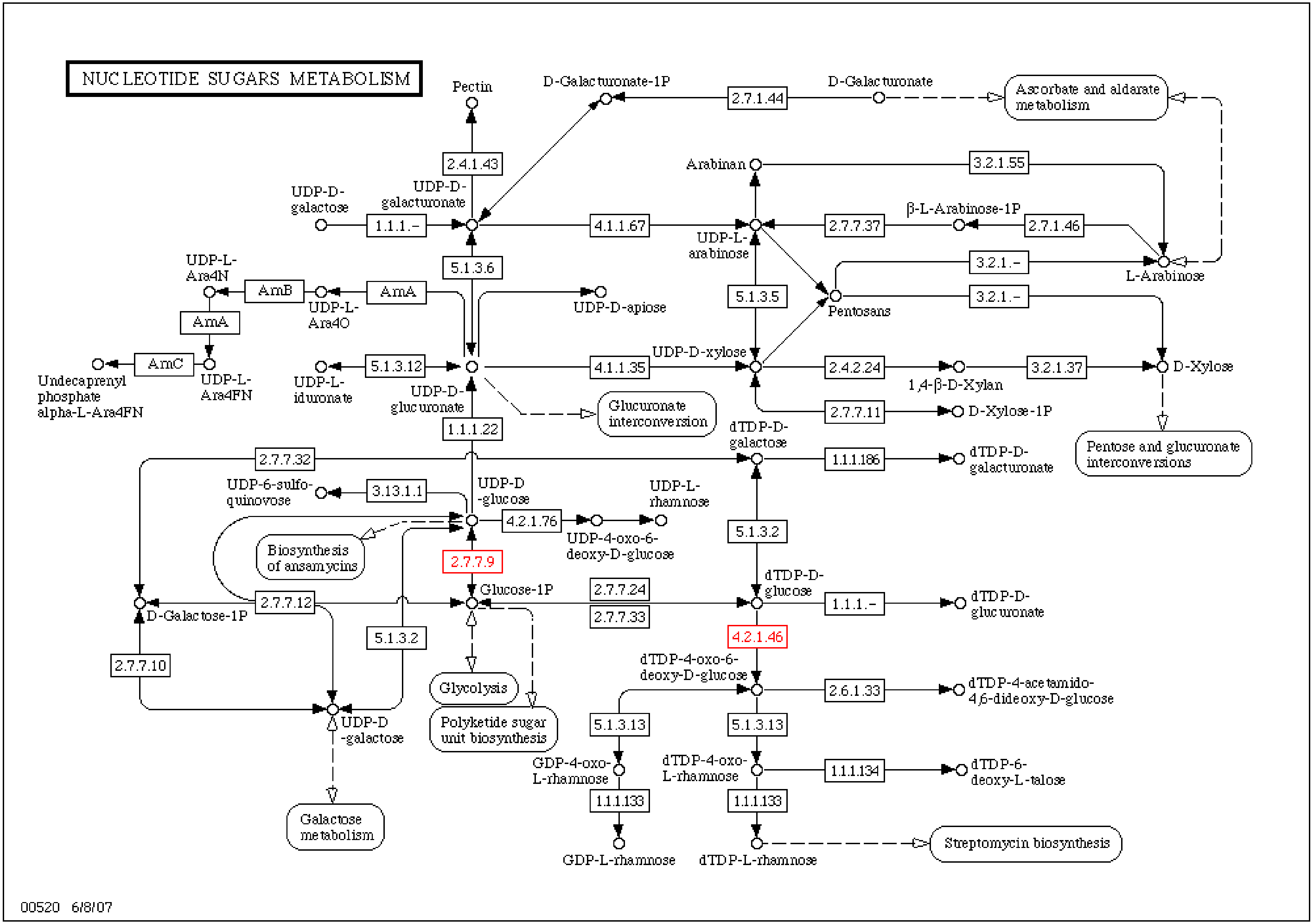
NUCLEOTIDE SUGARS METABOLISM
Pectin
D-Galacturonate-1P
2.7.1.44
D-Galacturonate
Ascorbate and aldarate metabolism
2.4.1.43
UDP-D-galactose
UDP-D-galacturonate
1.1.1.-
4.1.1.67
Arabinan
3.2.1.55
β-L-Arabinose-1P
2.7.7.37
2.7.1.46
UDP-L-arabinose
5.1.3.6
3.2.1.-
L-Arabinose
UDP-L-Ara4N
ArnB
ArnA
UDP-L-Ara4O
ArnA
UDP-D-apiose
5.1.3.5
Pentosans
3.2.1.-
ArnC
Undecaprenyl phosphate alpha-L-Ara4FN
UDP-L-Ara4FN
5.1.3.12
UDP-L-iduronate
4.1.1.35
UDP-D-xylose
2.4.2.24
1,4-β-D-Xylan
3.2.1.37
D-Xylose
UDP-D-glucuronate
Glucuronate interconversion
2.7.7.11
D-Xylose-1P
1.1.1.22
dTDP-D-galactose
Pentose and glucuronate interconversions
2.7.7.32
1.1.1.186
dTDP-D-galacturonate
UDP-6-sulfo-quinovose
3.13.1.1
UDP-D-glucose
UDP-L-rhamnose
4.2.1.76
UDP-4-oxo-6-deoxy-D-glucose
Biosynthesis of ansamycins
2.7.7.9
5.1.3.2
Glucose-1P
2.7.7.24
2.7.7.33
dTDP-D-glucose
1.1.1.-
dTDP-D-glucuronate
D-Galactose-1P
2.7.7.12
5.1.3.2
4.2.1.46
2.7.7.10
dTDP-4-oxo-6-deoxy-D-glucose
2.6.1.33
dTDP-4-acetamido-4,6-dideoxy-D-glucose
Glycolysis
Polyketide sugar unit biosynthesis
UDP-D-galactose
5.1.3.13
5.1.3.13
GDP-4-oxo-L-rhamnose
dTDP-4-oxo-L-rhamnose
1.1.1.134
dTDP-6-deoxy-L-talose
1.1.1.133
1.1.1.133
Galactose metabolism
GDP-L-rhamnose
dTDP-L-rhamnose
Streptomycin biosynthesis
00520 6/8/07

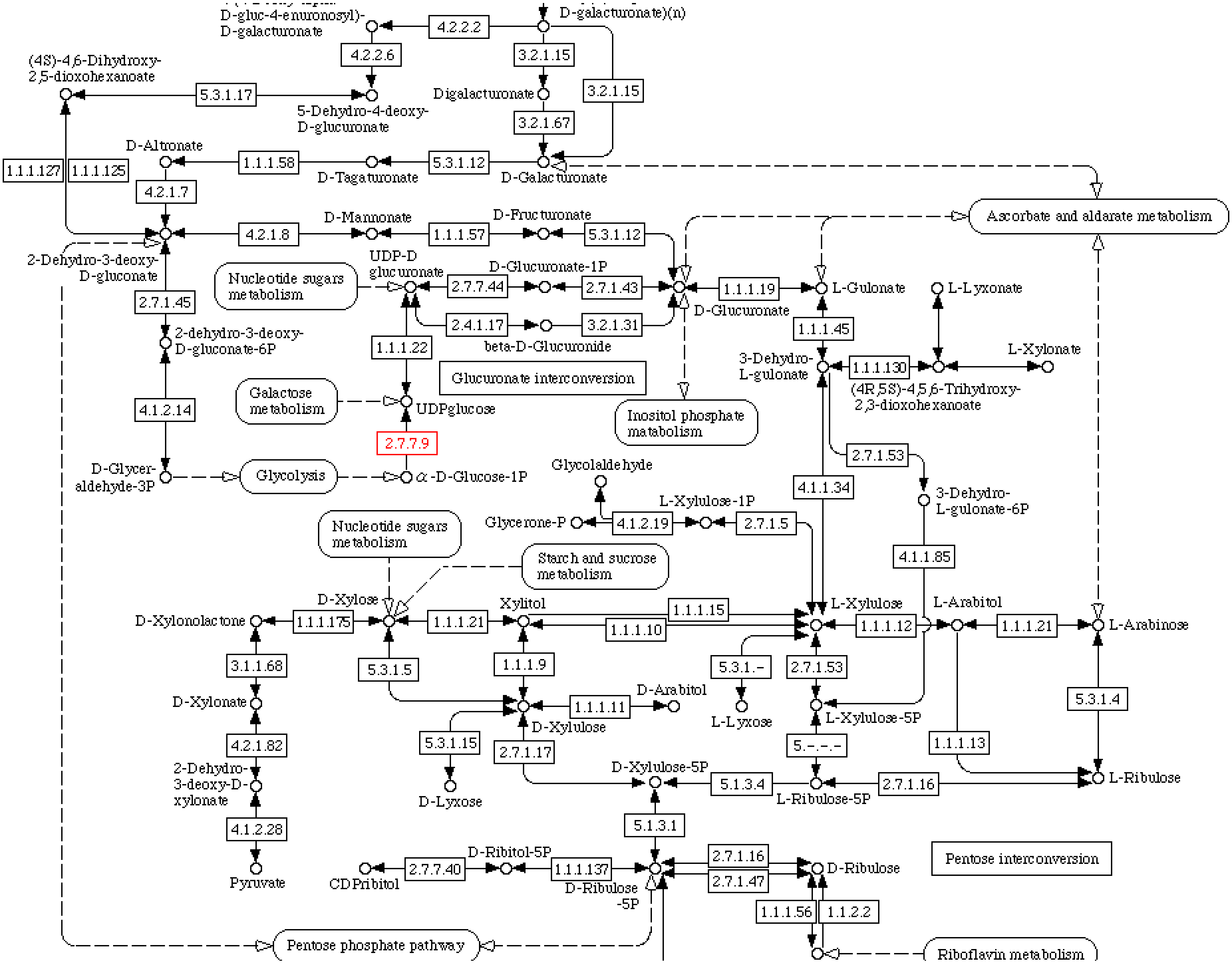
D-gluc-4-enuronosyl)-
D-galacturonate
D-galacturonate)(n)
4.2.2.2
4.2.2.6
3.2.1.15
3.2.1.15
(4S)-4,6-Dihydroxy-
2,5-dioxohexanoate
5.3.1.17
5-Dehydro-4-deoxy-
D-glucuronate
Digalacturonate
3.2.1.67
D-Altronate
1.1.1.58
5.3.1.12
D-Tagaturonate
D-Galacturonate
1.1.1.127
1.1.1.125
4.2.1.7
4.2.1.8
D-Mannonate
1.1.1.57
D-Fructuronate
5.3.1.12
Ascorbate and aldarate metabolism
2-Dehydro-3-deoxy-
D-gluconate
Nucleotide sugars
metabolism
UDP-D
glucuronate
D-Glucuronate-1P
2.7.7.44
2.7.1.43
1.1.1.19
L-Gulonate
L-Lyxonate
2.7.1.45
D-Glucuronate
2.4.1.17
3.2.1.31
1.1.1.45
2-dehydro-3-deoxy-
D-gluconate-6P
1.1.1.22
beta-D-Glucuronide
3-Dehydro-
L-gulonate
1.1.1.130
L-Xylonate
Glucuronate interconversion
(4R,5S)-4,5,6-Trihydroxy-
2,3-dioxohexanoate
Galactose
metabolism
UDPglucose
Inositol phosphate
matabolism
4.1.2.14
2.7.7.9
2.7.1.53
D-Glycer-
aldehyde-3P
Glycolysis
α-D-Glucose-1P
Glycolaldehyde
4.1.1.34
3-Dehydro-
L-gulonate-6P
L-Xylulose-1P
Glycerone-P
4.1.2.19
2.7.1.5
Nucleotide sugars
metabolism
Starch and sucrose
metabolism
4.1.1.85
D-Xylose
Xylitol
1.1.1.15
L-Xylulose
L-Arabitol
D-Xylonolactone
1.1.1.175
1.1.1.21
1.1.1.10
1.1.1.12
1.1.1.21
L-Arabinose
3.1.1.68
5.3.1.5
1.1.1.9
5.3.1.-
2.7.1.53
D-Xylonate
D-Arabitol
1.1.1.11
L-Lyxose
L-Xylulose-5P
5.3.1.4
D-Xylulose
4.2.1.82
5.3.1.15
2.7.1.17
5.-.-.-
1.1.1.13
2-Dehydro-
3-deoxy-D-
xylonate
D-Xylulose-5P
5.1.3.4
2.7.1.16
L-Ribulose
D-Lyxose
L-Ribulose-5P
5.1.3.1
4.1.2.28
Pentose interconversion
Pyruvate
D-Ribitol-5P
2.7.1.16
CDPribitol
2.7.7.40
1.1.1.137
D-Ribulose
2.7.1.47
D-Ribulose
-5P
1.1.1.56
1.1.2.2
Pentose phosphate pathway
Riboflavin metabolism

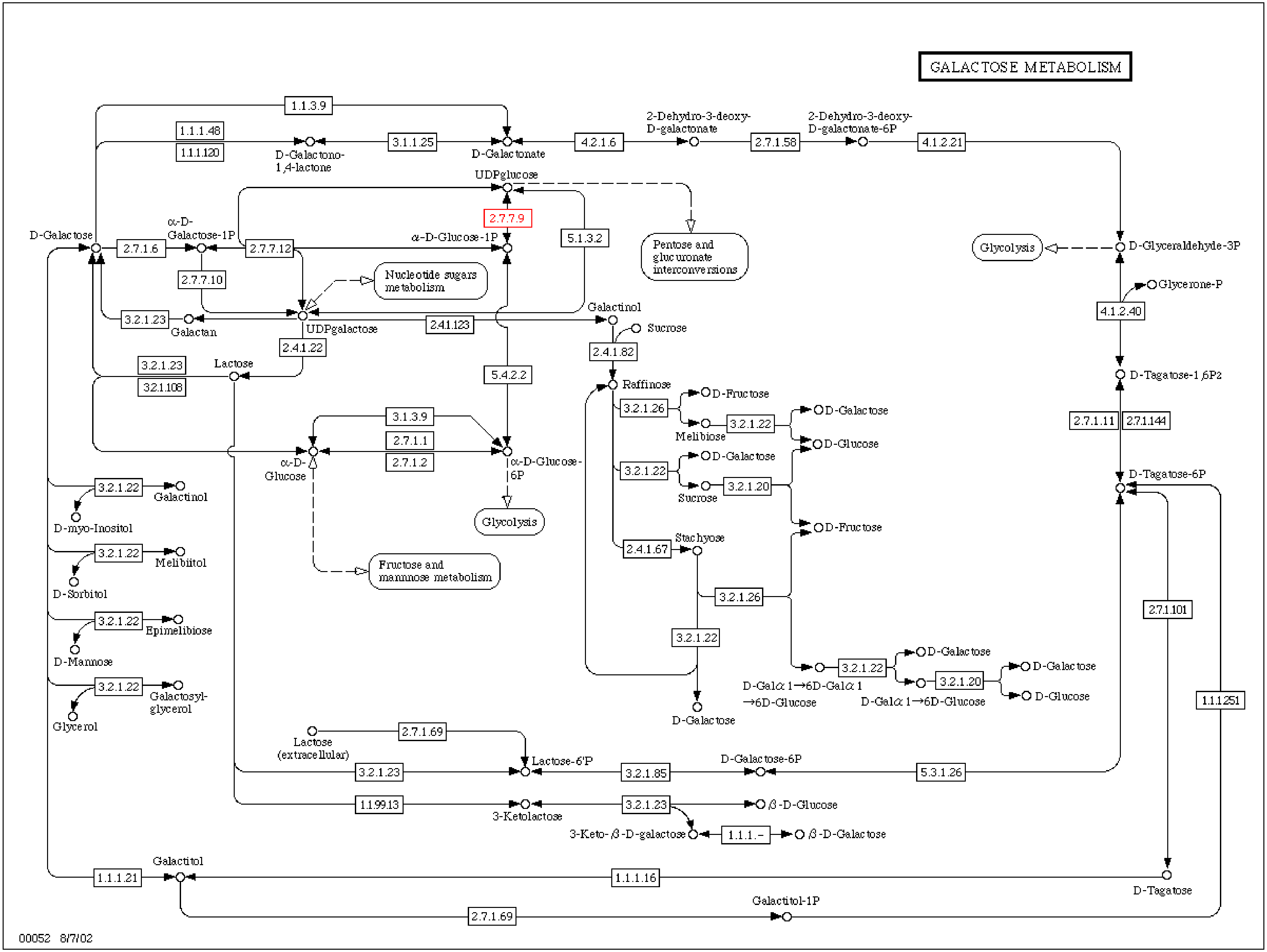
GALACTOSE METABOLISM
1.1.3.9
1.1.1.48
1.1.1.120
D-Galactono-1,4-lactone
3.1.1.25
D-Galactonate
4.2.1.6
2-Dehydro-3-deoxy-D-galactonate
2.7.1.58
2-Dehydro-3-deoxy-D-galactonate-6P
4.1.2.21
UDPglucose
2.7.7.9
5.1.3.2
Pentose and glucuronate interconversions
Glycolysis
D-Glyceraldehyde-3P
D-Galactose
α-D-Galactose-1P
2.7.1.6
2.7.7.12
α-D-Glucose-1P
2.7.7.10
Nucleotide sugars metabolism
Glycerone-P
4.1.2.40
3.2.1.23
Galactan
UDPgalactose
2.4.1.123
Galactinol
Sucrose
2.4.1.22
2.4.1.82
3.2.1.23
3.2.1.108
Lactose
5.4.2.2
D-Tagatose-1,6P2
Raffinose
D-Fructose
3.2.1.26
3.2.1.22
D-Galactose
Melibiose
D-Glucose
2.7.1.11
2.7.1.144
3.1.3.9
2.7.1.1
2.7.1.2
α-D-Glucose
α-D-Glucose-6P
3.2.1.22
D-Galactose
3.2.1.20
Sucrose
D-Tagatose-6P
3.2.1.22
Galactinol
D-myo-Inositol
Glycolysis
D-Fructose
Stachyose
2.4.1.67
3.2.1.22
Melibiitol
D-Sorbitol
Fructose and mannose metabolism
3.2.1.26
2.7.1.101
3.2.1.22
Epimelibiose
D-Mannose
3.2.1.22
3.2.1.22
D-Galactose
D-Galactose
3.2.1.22
Galactosyl-glycerol
Glycerol
D-Galα1→6D-Galα1→6D-Glucose
3.2.1.20
D-Galα1→6D-Glucose
D-Glucose
D-Galactose
1.1.1.251
Lactose (extracellular)
2.7.1.69
Lactose-6'P
3.2.1.23
3.2.1.85
D-Galactose-6P
5.3.1.26
1.1.99.13
3-Ketolactose
3.2.1.23
β-D-Glucose
3-Keto-β-D-galactose
1.1.1.-
β-D-Galactose
Galactitol
1.1.1.21
1.1.1.16
D-Tagatose
2.7.1.69
Galactitol-1P
00052 8/7/02

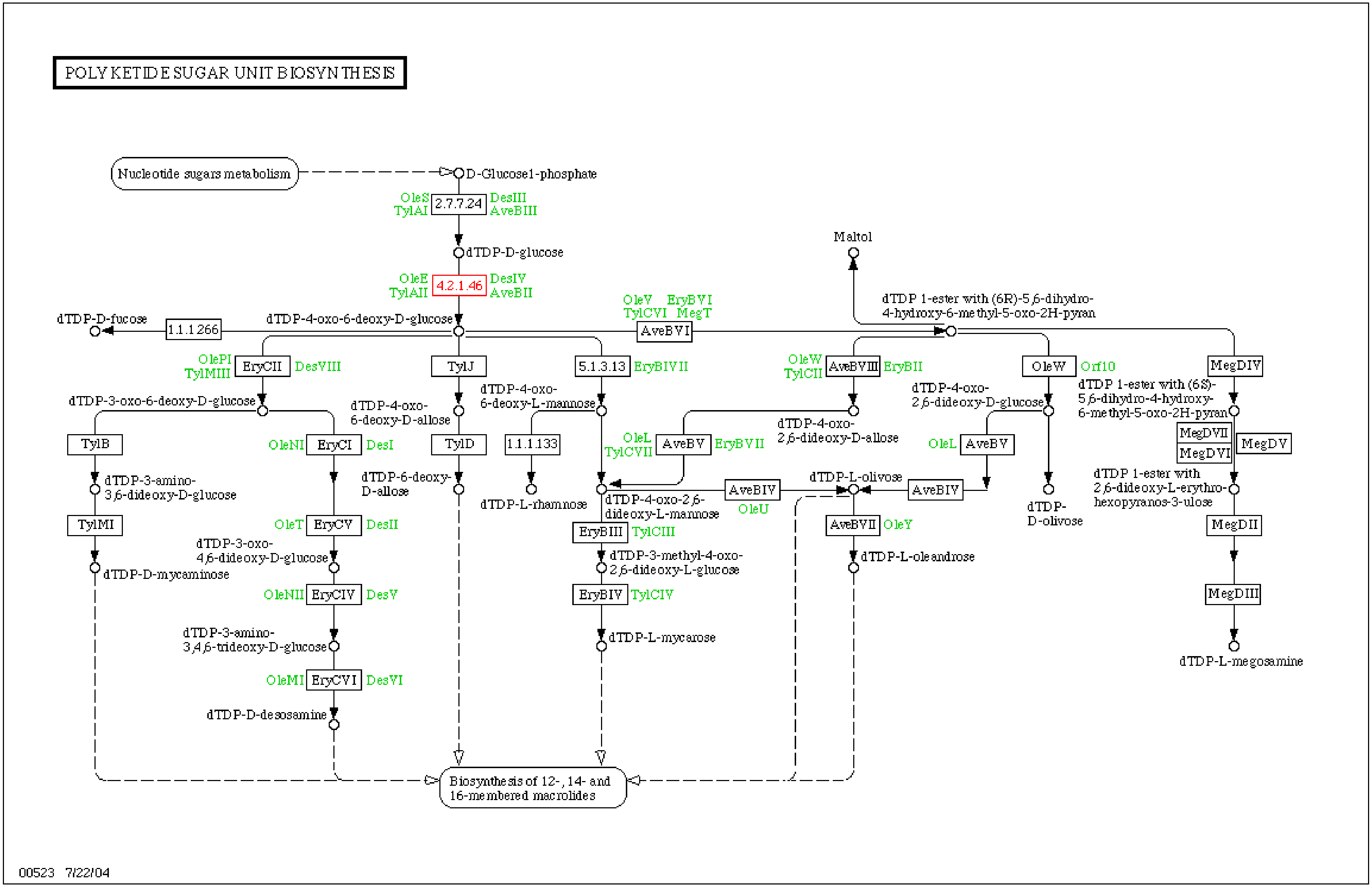
POLYKETIDE SUGAR UNIT BIOSYNTHESIS
Nucleotide sugars metabolism
D-Glucose1-phosphate
OleS
TylAI
2.7.7.24
DesIII
AveBIII
dTDP-D-glucose
OleE
TylAII
4.2.1.46
DesIV
AveBII
Maltol
dTDP-D-fucose
1.1.1.266
dTDP-4-oxo-6-deoxy-D-glucose
OleV EryBVI
TylCVI MegT
AveBVI
dTDP 1-ester with (6R)-5,6-dihydro-4-hydroxy-6-methyl-5-oxo-2H-pyran
OlePI
TylMIII
EryCII
DesVIII
TylJ
5.1.3.13
EryBVII
OleW
TylCII
AveBVIII
EryBII
OleW
Orf10
MegDIV
dTDP-3-oxo-6-deoxy-D-glucose
dTDP-4-oxo-6-deoxy-D-allose
dTDP-4-oxo-6-deoxy-L-mannose
dTDP-4-oxo-2,6-dideoxy-D-glucose
dTDP 1-ester with (6S)-5,6-dihydro-4-hydroxy-6-methyl-5-oxo-2H-pyran
TylB
OleNI
EryCI
DesI
TylD
1.1.1.133
OleL
TylCVII
AveBV
EryBVII
dTDP-4-oxo-2,6-dideoxy-D-allose
OleL
AveBV
MegDVII
MegDVI
MegDV
dTDP-3-amino-3,6-dideoxy-D-glucose
dTDP-6-deoxy-D-allose
dTDP-L-rhamnose
dTDP-4-oxo-2,6-dideoxy-L-mannose
AveBIV
OleU
dTDP-L-olivose
AveBIV
dTDP-D-olivose
dTDP 1-ester with 2,6-dideoxy-L-erythro-hexopyranos-3-ulose
TylMI
OleT
EryCV
DesII
EryBIII
TylCIII
AveBVII
OleY
MegDII
dTDP-3-oxo-4,6-dideoxy-D-glucose
dTDP-D-mycaminose
dTDP-3-methyl-4-oxo-2,6-dideoxy-L-glucose
dTDP-L-oleandrose
OleNII
EryCIV
DesV
EryBIV
TylCIV
MegDIII
dTDP-3-amino-3,4,6-trideoxy-D-glucose
dTDP-L-mycarose
dTDP-L-megosamine
OleMI
EryCVI
DesVI
dTDP-D-desosamine
Biosynthesis of 12-, 14- and 16-membered macrolides
00523 7/22/04

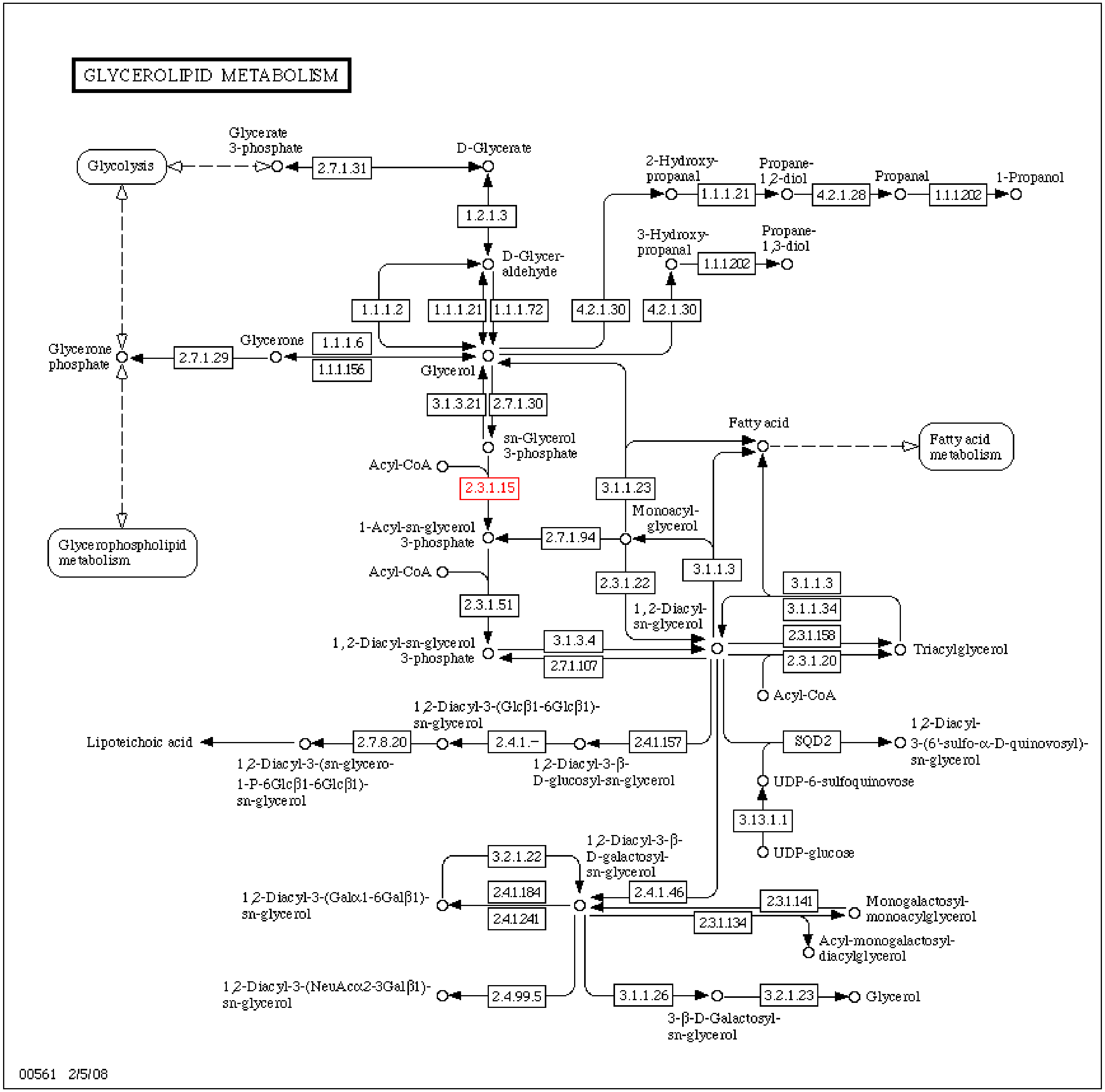
GLYCEROLIPID METABOLISM
Glycolysis
Glycerate 3-phosphate
2.7.1.31
D-Glycerate
1.2.1.3
D-Glyceraldehyde
1.1.1.2
1.1.1.21
1.1.1.72
2-Hydroxypropanal
1.1.1.21
Propane-1,2-diol
4.2.1.28
Propanal
1.1.1.202
1-Propanol
3-Hydroxypropanal
1.1.1.202
Propane-1,3-diol
4.2.1.30
4.2.1.30
Glycerone phosphate
2.7.1.29
Glycerone
1.1.1.6
1.1.1.156
Glycerol
3.1.3.21
2.7.1.30
sn-Glycerol 3-phosphate
Acyl-CoA
2.3.1.15
Fatty acid
Fatty acid metabolism
3.1.1.23
Monoacylglycerol
Glycerophospholipid metabolism
1-Acyl-sn-glycerol 3-phosphate
2.7.1.94
3.1.1.3
Acyl-CoA
2.3.1.22
3.1.1.3
3.1.1.34
2.3.1.51
1,2-Diacyl-sn-glycerol
2.3.1.158
1,2-Diacyl-sn-glycerol 3-phosphate
3.1.3.4
2.7.1.107
2.3.1.20
Triacylglycerol
Acyl-CoA
1,2-Diacyl-3-(Glcβ1-6Glcβ1)-sn-glycerol
Lipoteichoic acid
2.7.8.20
2.4.1.-
2.4.1.157
SQD2
1,2-Diacyl-3-(6'-sulfo-α-D-quinovosyl)-sn-glycerol
1,2-Diacyl-3-(sn-glycero-1-P-6Glcβ1-6Glcβ1)-sn-glycerol
1,2-Diacyl-3-β-D-glucosyl-sn-glycerol
UDP-6-sulfoquinovose
3.13.1.1
UDP-glucose
1,2-Diacyl-3-β-D-galactosyl-sn-glycerol
3.2.1.22
2.4.1.184
2.4.1.241
2.4.1.46
2.3.1.141
2.3.1.134
1,2-Diacyl-3-(Galα1-6Galβ1)-sn-glycerol
Monogalactosyl-monoacylglycerol
Acyl-monogalactosyl-diacylglycerol
1,2-Diacyl-3-(NeuAcα2-3Galβ1)-sn-glycerol
2.4.99.5
3.1.1.26
3.2.1.23
Glycerol
3-β-D-Galactosyl-sn-glycerol
00561 2/5/08

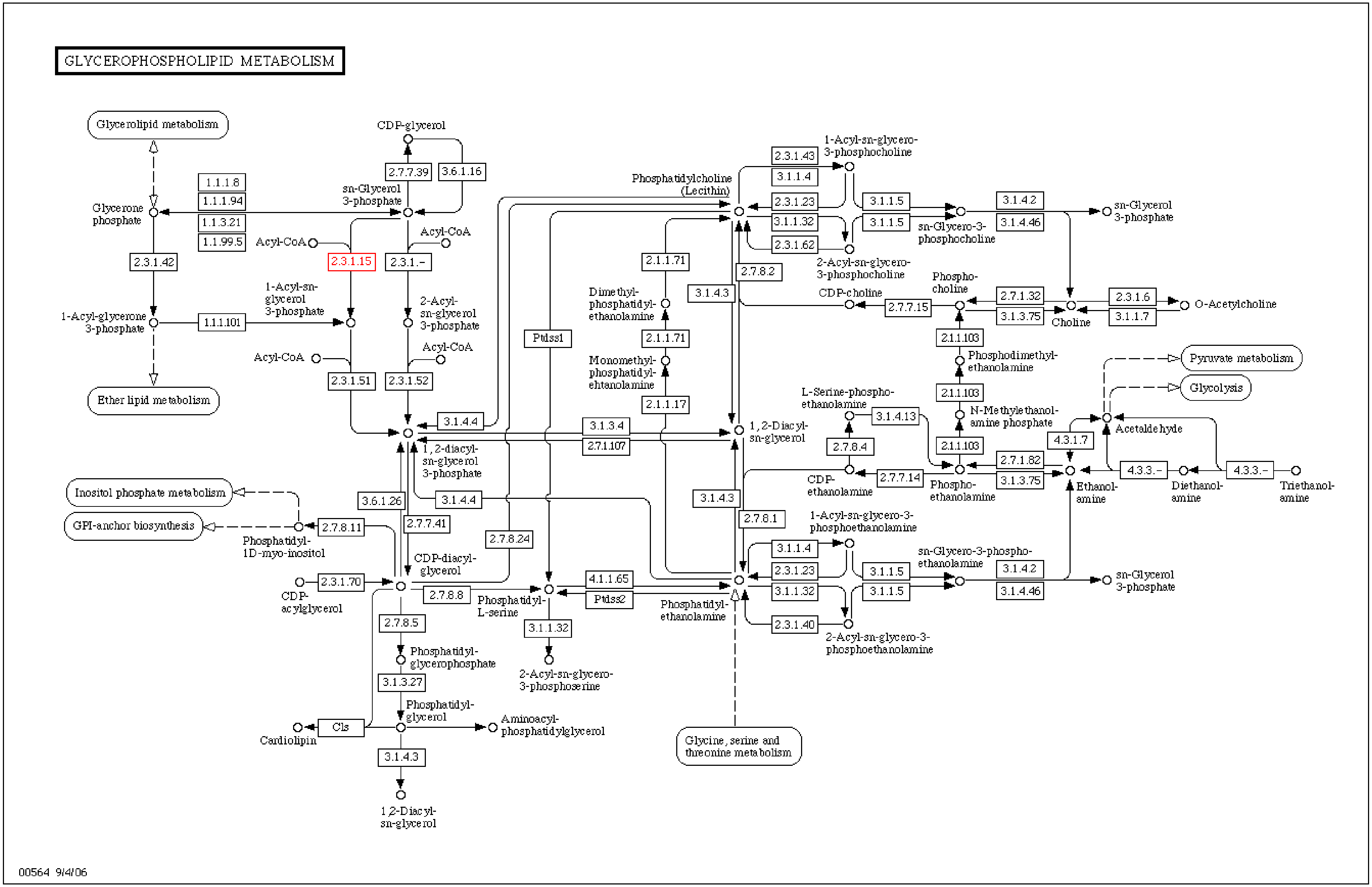

GLYCEROPHOSPHOLIPID METABOLISM
Glycerolipid metabolism
Glycerone phosphate
1.1.1.8
1.1.1.94
1.1.3.21
1.1.99.5
2.3.1.42
1-Acyl-glycerone 3-phosphate
1.1.1.101
Ether lipid metabolism
CDP-glycerol
2.7.7.39
3.6.1.16
sn-Glycerol 3-phosphate
Acyl-CoA
2.3.1.15
2.3.1.-
Acyl-CoA
1-Acyl-sn-glycerol 3-phosphate
2-Acyl-sn-glycerol 3-phosphate
Acyl-CoA
2.3.1.51
Acyl-CoA
2.3.1.52
3.1.4.4
3.1.3.4
2.7.1.107
1,2-diacyl-sn-glycerol 3-phosphate
3.6.1.26
3.1.4.4
2.7.7.41
Inositol phosphate metabolism
GPI-anchor biosynthesis
2.7.8.11
Phosphatidyl-1D-myo-inositol
2.7.8.24
CDP-diacyl-glycerol
2.3.1.70
CDP-acylglycerol
2.7.8.8
Phosphatidyl-L-serine
4.1.1.65
Ptdss2
3.1.1.32
2-Acyl-sn-glycero-3-phosphoserine
2.7.8.5
Phosphatidyl-glycerophosphate
3.1.3.27
Phosphatidyl-glycerol
Cls
Cardiolipin
Aminoacyl-phosphatidylglycerol
3.1.4.3
1,2-Diacyl-sn-glycerol
Ptdss1
Phosphatidylcholine (Lecithin)
2.1.1.71
Dimethyl-phosphatidyl-ethanolamine
2.1.1.71
Monomethyl-phosphatidyl-ehtanolamine
2.1.1.17
3.1.4.3
2.7.8.2
2.3.1.43
3.1.1.4
2.3.1.23
3.1.1.32
2.3.1.62
1-Acyl-sn-glycero-3-phosphocholine
2-Acyl-sn-glycero-3-phosphocholine
3.1.1.5
3.1.1.5
sn-Glycero-3-phosphocholine
3.1.4.2
3.1.4.46
sn-Glycerol 3-phosphate
CDP-choline
2.7.7.15
Phospho-choline
2.7.1.32
3.1.3.75
Choline
2.3.1.6
3.1.1.7
O-Acetylcholine
2.1.1.103
Phosphodimethyl-ethanolamine
2.1.1.103
N-Methylethanol-amine phosphate
2.1.1.103
Pyruvate metabolism
Glycolysis
Acetaldehyde
4.3.1.7
L-Serine-phospho-ethanolamine
3.1.4.13
2.7.8.4
1,2-Diacyl-sn-glycerol
3.1.4.3
2.7.8.1
CDP-ethanolamine
2.7.7.14
Phospho-ethanolamine
2.7.1.82
3.1.3.75
Ethanol-amine
4.3.3.-
Diethanol-amine
4.3.3.-
Triethanol-amine
1-Acyl-sn-glycero-3-phosphoethanolamine
3.1.1.4
2.3.1.23
3.1.1.32
2.3.1.40
Phosphatidyl-ethanolamine
2-Acyl-sn-glycero-3-phosphoethanolamine
3.1.1.5
3.1.1.5
sn-Glycero-3-phospho-ethanolamine
3.1.4.2
3.1.4.46
sn-Glycerol 3-phosphate
Glycine, serine and threonine metabolism
00564 9/4/06

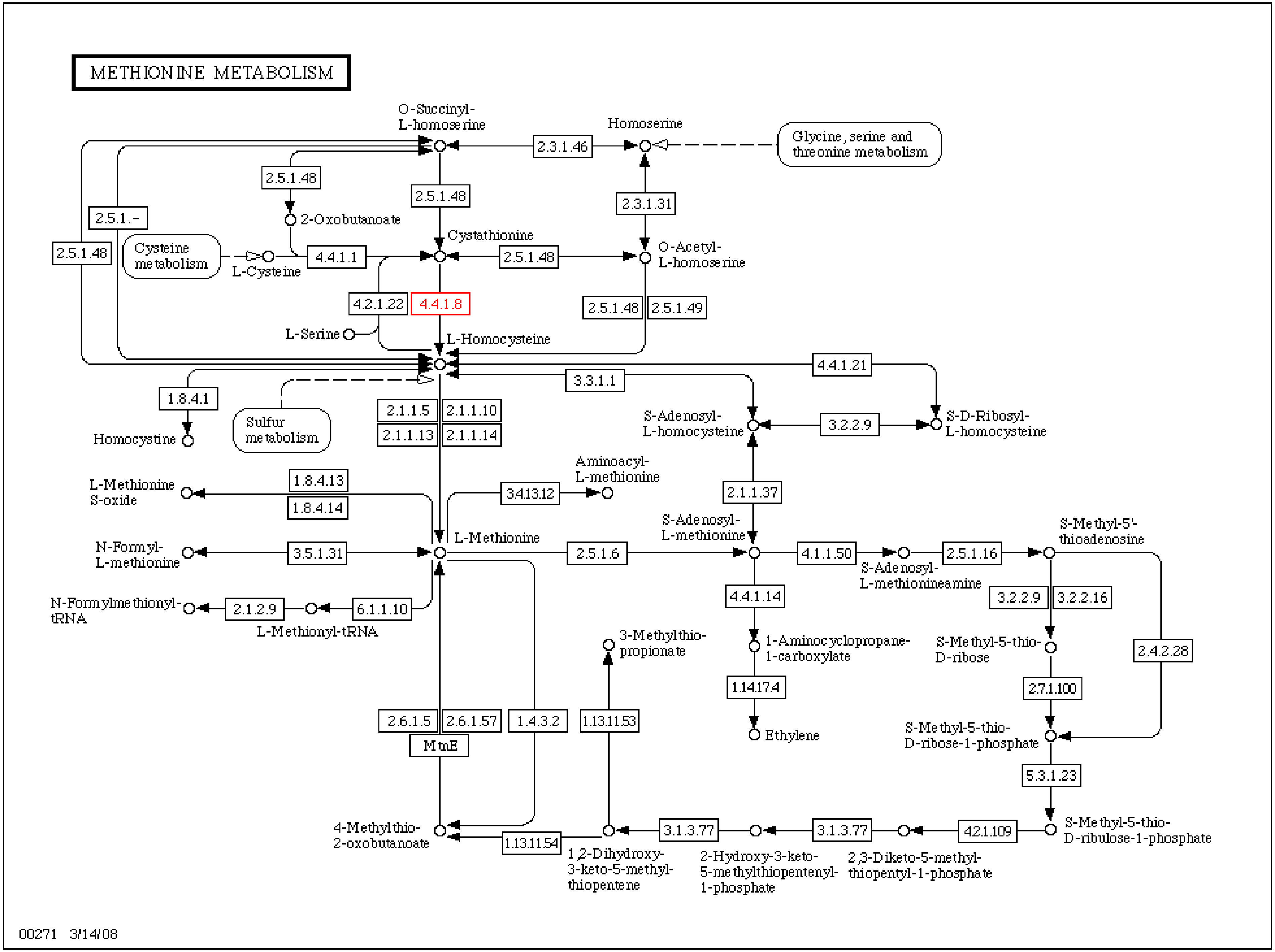
METHIONINE METABOLISM
O-Succinyl-
L-homoserine
Homoserine
Glycine, serine and threonine metabolism
2.3.1.46
2.5.1.48
2.5.1.48
2.5.1.-
2.3.1.31
2-Oxobutanoate
Cystathionine
2.5.1.48
Cysteine metabolism
L-Cysteine
4.4.1.1
2.5.1.48
O-Acetyl-
L-homoserine
4.2.1.22
4.4.1.8
2.5.1.48
2.5.1.49
L-Serine
L-Homocysteine
4.4.1.21
3.3.1.1
1.8.4.1
Sulfur metabolism
2.1.1.5
2.1.1.10
2.1.1.13
2.1.1.14
S-Adenosyl-
L-homocysteine
3.2.2.9
S-D-Ribosyl-
L-homocysteine
Homocystine
Aminoacyl-
L-methionine
L-Methionine
S-oxide
1.8.4.13
1.8.4.14
3.4.13.12
2.1.1.37
S-Adenosyl-
L-methionine
S-Methyl-5'-
thioadenosine
N-Formyl-
L-methionine
3.5.1.31
L-Methionine
2.5.1.6
4.1.1.50
2.5.1.16
S-Adenosyl-
L-methionineamine
N-Formylmethionyl-
tRNA
2.1.2.9
6.1.1.10
4.4.1.14
3.2.2.9
3.2.2.16
L-Methionyl-tRNA
3-Methylthio-
propionate
1-Aminocyclopropane-
1-carboxylate
S-Methyl-5-thio-
D-ribose
2.4.2.28
1.14.17.4
2.7.1.100
2.6.1.5
2.6.1.57
1.4.3.2
1.13.11.53
Ethylene
S-Methyl-5-thio-
D-ribose-1-phosphate
MtnE
5.3.1.23
4-Methylthio-
2-oxobutanoate
1.13.11.54
3.1.3.77
3.1.3.77
4.2.1.109
S-Methyl-5-thio-
D-ribulose-1-phosphate
1,2-Dihydroxy-
3-keto-5-methyl-
thiopentene
2-Hydroxy-3-keto-
5-methylthiopentenyl-
1-phosphate
2,3-Diketo-5-methyl-
thiopentyl-1-phosphate
00271 3/14/08

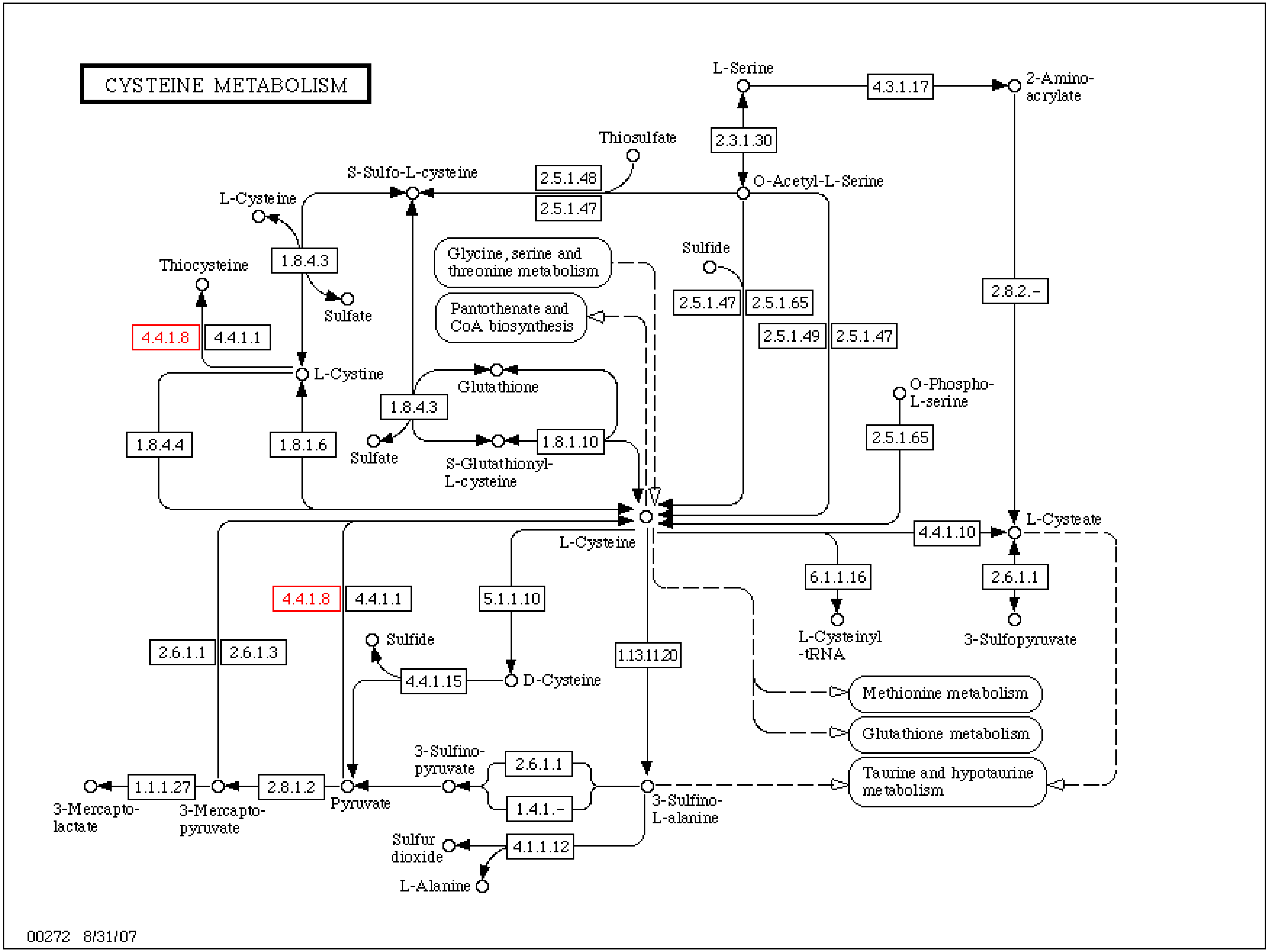
CYSTEINE METABOLISM
L-Serine
4.3.1.17
2-Amino-acrylate
2.3.1.30
Thiosulfate
S-Sulfo-L-cysteine
2.5.1.48
O-Acetyl-L-Serine
2.5.1.47
L-Cysteine
1.8.4.3
Thiocysteine
Sulfate
4.4.1.8
4.4.1.1
Glycine, serine and threonine metabolism
Pantothenate and CoA biosynthesis
Sulfide
2.5.1.47
2.5.1.65
2.5.1.49
2.5.1.47
2.8.2.-
L-Cystine
Glutathione
1.8.4.3
O-Phospho-L-serine
1.8.4.4
1.8.1.6
Sulfate
1.8.1.10
S-Glutathionyl-L-cysteine
2.5.1.65
L-Cysteine
4.4.1.10
L-Cysteate
6.1.1.16
2.6.1.1
4.4.1.8
4.4.1.1
5.1.1.10
L-Cysteinyl-tRNA
3-Sulfopyruvate
2.6.1.1
2.6.1.3
Sulfide
1.13.11.20
4.4.1.15
D-Cysteine
Methionine metabolism
Glutathione metabolism
3-Sulfino-pyruvate
2.6.1.1
Taurine and hypotaurine metabolism
1.1.1.27
2.8.1.2
1.4.1.-
3-Mercapto-lactate
3-Mercapto-pyruvate
Pyruvate
3-Sulfino-L-alanine
Sulfur dioxide
4.1.1.12
L-Alanine
00272 8/31/07

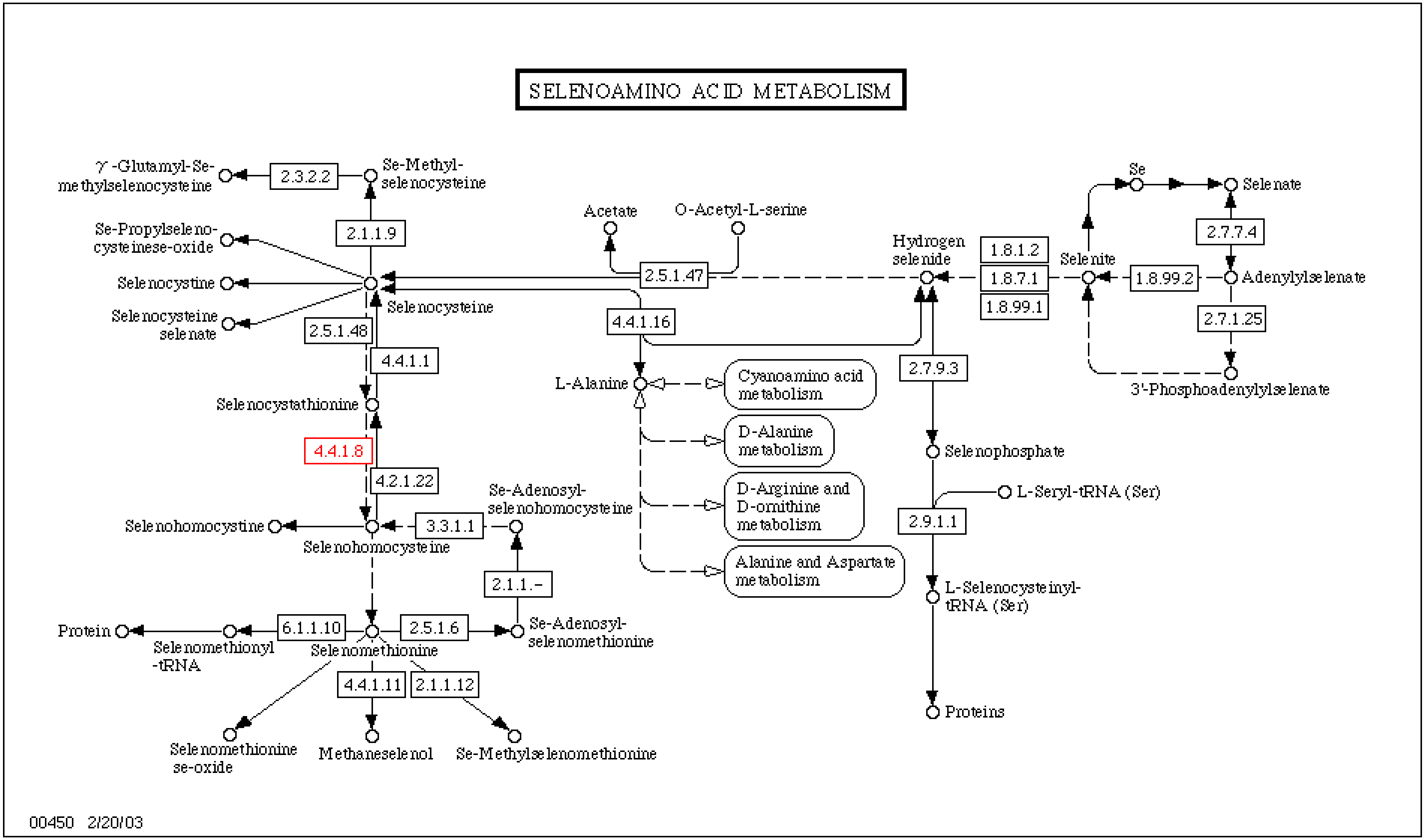
SELENOAMINO ACID METABOLISM
γ-Glutamyl-Se-methylselenocysteine
2.3.2.2
Se-Methyl-selenocysteine
2.1.1.9
Se-Propylseleno-cysteinese-oxide
Selenocystine
Selenocysteine selenate
Selenocysteine
2.5.1.48
4.4.1.1
Selenocystathionine
4.4.1.8
4.2.1.22
Selenohomocystine
Selenohomocysteine
3.3.1.1
Se-Adenosyl-selenohomocysteine
2.1.1.-
Protein
Selenomethionyl-tRNA
6.1.1.10
Selenomethionine
2.5.1.6
Se-Adenosyl-selenomethionine
4.4.1.11
2.1.1.12
Selenomethionine se-oxide
Methaneselenol
Se-Methylselenomethionine
Acetate
O-Acetyl-L-serine
2.5.1.47
4.4.1.16
L-Alanine
Cyanoamino acid metabolism
D-Alanine metabolism
D-Arginine and D-ornithine metabolism
Alanine and Aspartate metabolism
Hydrogen selenide
1.8.1.2
1.8.7.1
1.8.99.1
2.7.9.3
Selenophosphate
L-Seryl-tRNA (Ser)
2.9.1.1
L-Selenocysteinyl-tRNA (Ser)
Proteins
Se
Selenate
Selenite
2.7.7.4
1.8.99.2
Adenylylselenate
2.7.1.25
3'-Phosphoadenylylselenate
00450 2/20/03

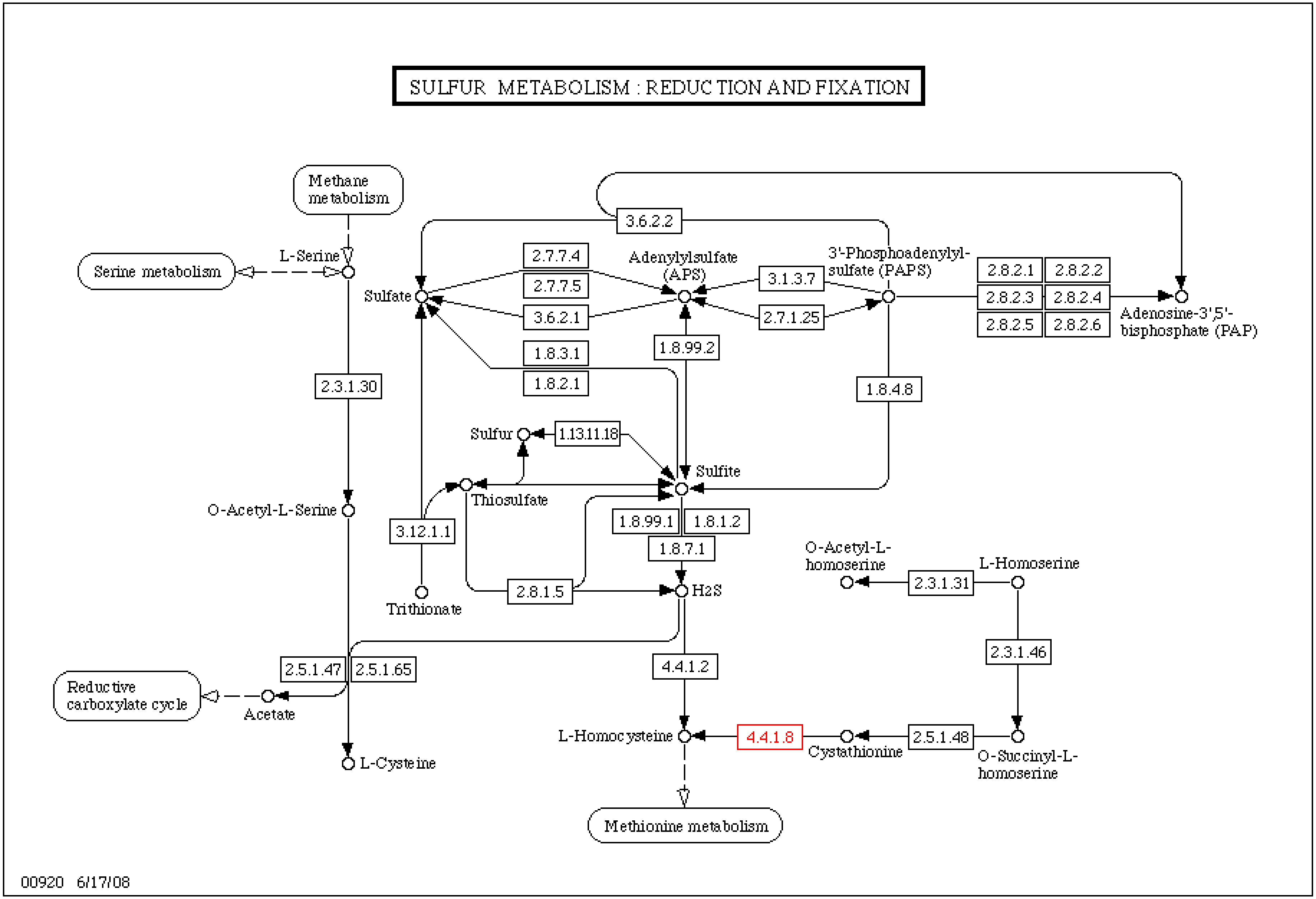
SULFUR METABOLISM : REDUCTION AND FIXATION
Methane metabolism
Serine metabolism
L-Serine
Sulfate
2.7.7.4
2.7.7.5
3.6.2.1
3.6.2.2
Adenylylsulfate (APS)
3.1.3.7
2.7.1.25
3'-Phosphoadenylyl-sulfate (PAPS)
2.8.2.1
2.8.2.2
2.8.2.3
2.8.2.4
2.8.2.5
2.8.2.6
Adenosine-3',5'-bisphosphate (PAP)
1.8.3.1
1.8.2.1
1.8.99.2
1.8.4.8
2.3.1.30
Sulfur
1.13.11.18
Sulfite
Thiosulfate
O-Acetyl-L-Serine
3.12.1.1
1.8.99.1
1.8.1.2
1.8.7.1
Trithionate
2.8.1.5
H2S
O-Acetyl-L-homoserine
2.3.1.31
L-Homoserine
2.3.1.46
2.5.1.47
2.5.1.65
4.4.1.2
Reductive carboxylate cycle
Acetate
L-Homocysteine
4.4.1.8
Cystathionine
2.5.1.48
O-Succinyl-L-homoserine
L-Cysteine
Methionine metabolism
00920 6/17/08

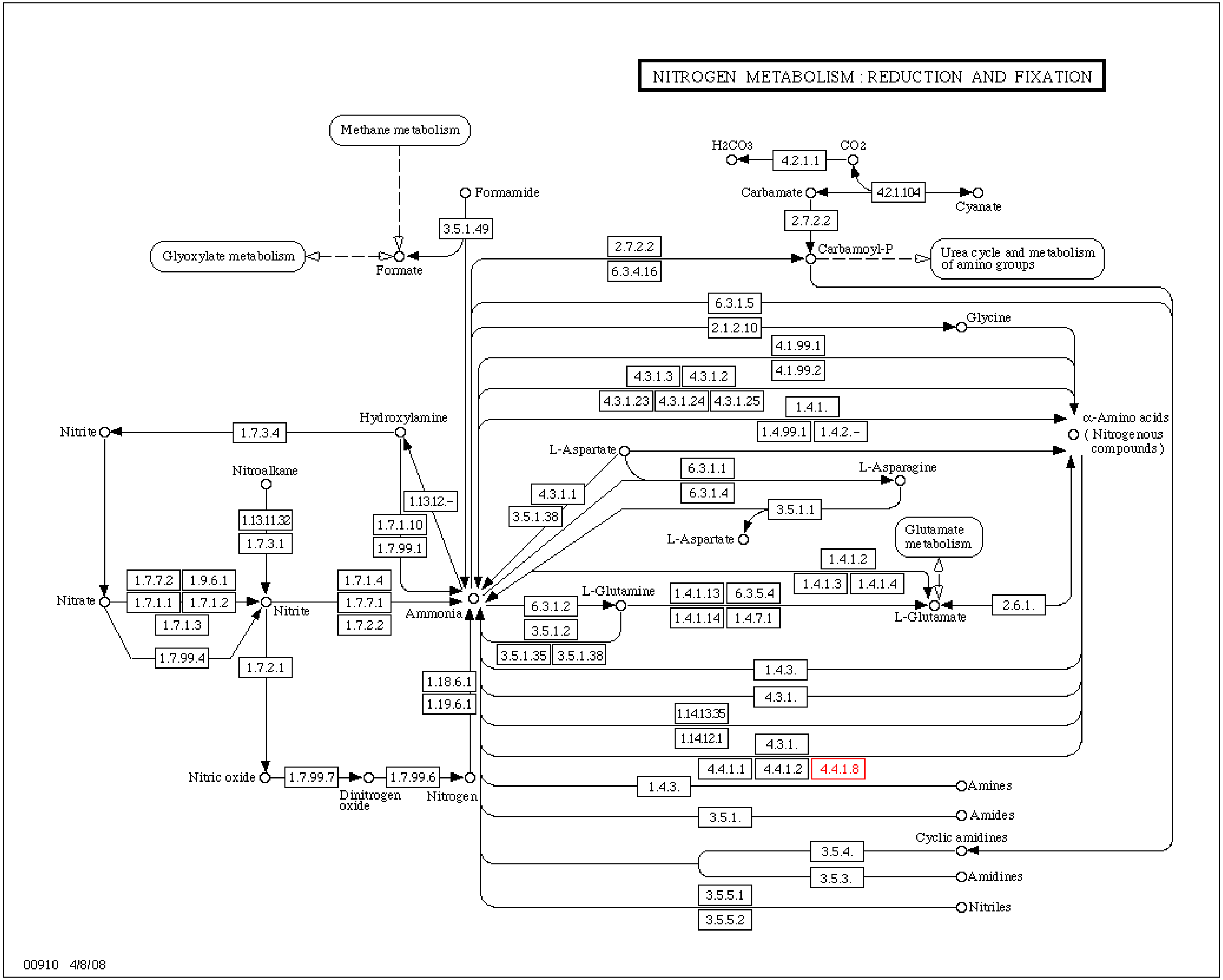

NITROGEN METABOLISM : REDUCTION AND FIXATION
Methane metabolism
Glyoxylate metabolism
Formate
Formamide
3.5.1.49
H2CO3
CO2
4.2.1.1
Carbamate
4.2.1.104
Cyanate
2.7.2.2
Carbamoyl-P
Urea cycle and metabolism of amino groups
2.7.2.2
6.3.4.16
6.3.1.5
2.1.2.10
Glycine
4.1.99.1
4.1.99.2
4.3.1.3
4.3.1.2
4.3.1.23
4.3.1.24
4.3.1.25
1.4.1.
1.4.99.1
1.4.2.-
α-Amino acids (Nitrogenous compounds)
Nitrite
1.7.3.4
Hydroxylamine
Nitroalkane
1.13.11.32
1.7.3.1
1.13.12.-
1.7.1.10
1.7.99.1
L-Aspartate
6.3.1.1
6.3.1.4
L-Asparagine
4.3.1.1
3.5.1.38
3.5.1.1
L-Aspartate
Glutamate metabolism
1.4.1.2
1.4.1.3
1.4.1.4
Nitrate
1.7.7.2
1.9.6.1
1.7.1.1
1.7.1.2
1.7.1.3
Nitrite
1.7.1.4
1.7.7.1
1.7.2.2
Ammonia
6.3.1.2
L-Glutamine
3.5.1.2
1.4.1.13
6.3.5.4
1.4.1.14
1.4.7.1
L-Glutamate
2.6.1.
1.7.99.4
1.7.2.1
3.5.1.35
3.5.1.38
1.18.6.1
1.19.6.1
1.4.3.
4.3.1.
1.14.13.35
1.14.12.1
4.3.1.
4.4.1.1
4.4.1.2
4.4.1.8
Nitric oxide
1.7.99.7
Dinitrogen oxide
1.7.99.6
Nitrogen
1.4.3.
Amines
3.5.1.
Amides
Cyclic amidines
3.5.4.
3.5.3.
Amidines
3.5.5.1
3.5.5.2
Nitriles
00910 4/8/08

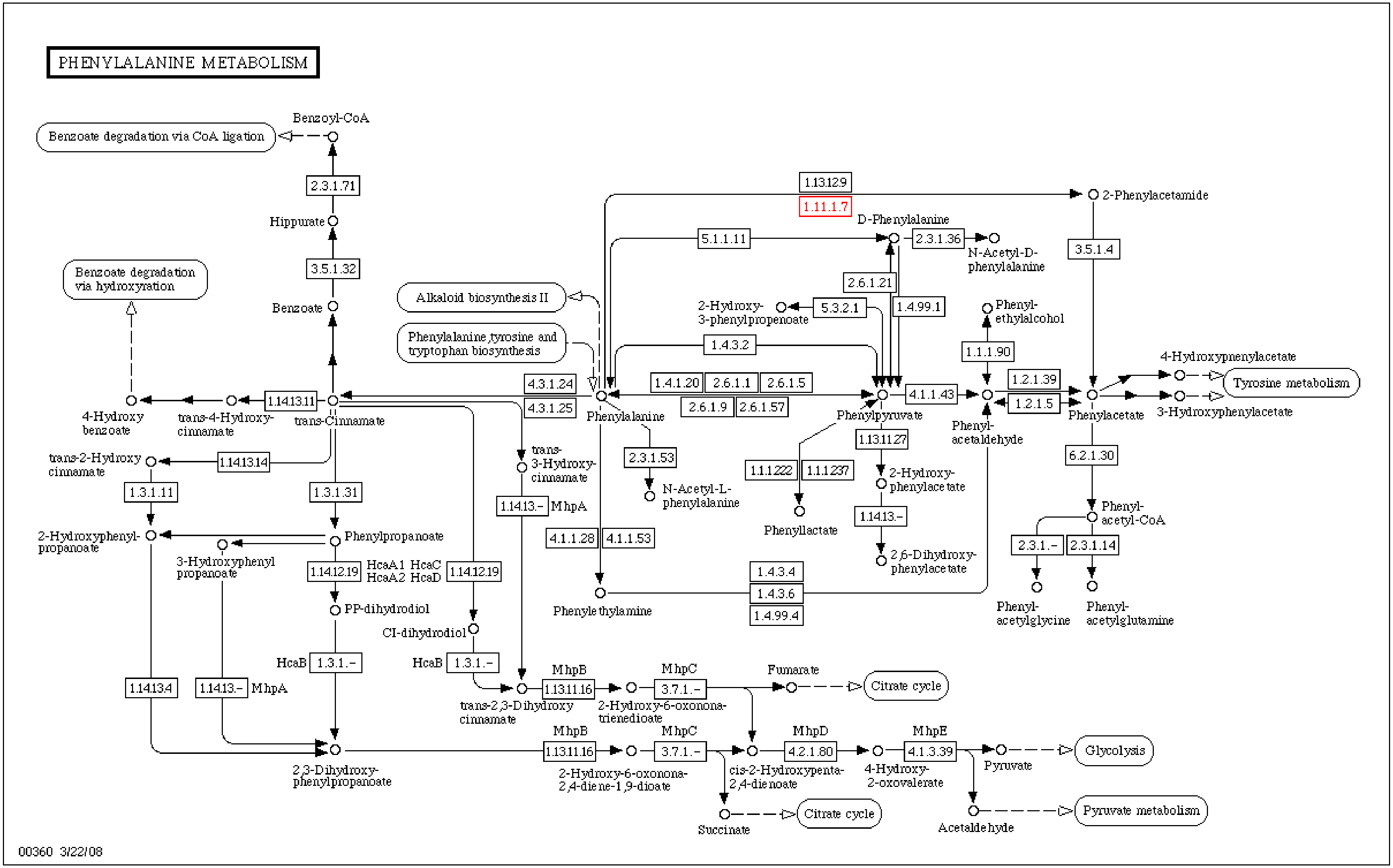
PHENYLALANINE METABOLISM
Benzoyl-CoA
Benzoate degradation via CoA ligation
2.3.1.71
Hippurate
3.5.1.32
Benzoate degradation via hydroxyration
Benzoate
4-Hydroxy benzoate
trans-4-Hydroxy-cinnamate
1.14.13.11
trans-Cinnamate
trans-2-Hydroxy cinnamate
1.14.13.14
1.3.1.11
1.3.1.31
2-Hydroxyphenyl-propanoate
3-Hydroxyphenyl propanoate
Phenylpropanoate
1.14.12.19
HcaA1 HcaC
HcaA2 HcaD
1.14.12.19
PP-dihydrodiol
CI-dihydrodiol
HcaB
1.3.1.-
HcaB
1.3.1.-
1.14.13.4
1.14.13.- MhpA
2,3-Dihydroxy-phenylpropanoate
trans-2,3-Dihydroxy cinnamate
MhpB
1.13.11.16
2-Hydroxy-6-oxonona-trienedioate
MhpC
3.7.1.-
Fumarate
Citrate cycle
MhpB
1.13.11.16
2-Hydroxy-6-oxonona-2,4-diene-1,9-dioate
MhpC
3.7.1.-
cis-2-Hydroxypenta-2,4-dienoate
MhpD
4.2.1.80
4-Hydroxy-2-oxovalerate
MhpE
4.1.3.39
Pyruvate
Glycolysis
Succinate
Citrate cycle
Acetaldehyde
Pyruvate metabolism
Alkaloid biosynthesis II
Phenylalanine, tyrosine and tryptophan biosynthesis
4.3.1.24
4.3.1.25
trans-3-Hydroxy-cinnamate
1.14.13.- MhpA
Phenylalanine
1.13.12.9
1.11.1.7
2-Phenylacetamide
5.1.1.11
D-Phenylalanine
2.3.1.36
N-Acetyl-D-phenylalanine
2.6.1.21
2-Hydroxy-3-phenylpropenoate
5.3.2.1
1.4.99.1
1.4.3.2
1.4.1.20
2.6.1.1
2.6.1.5
2.6.1.9
2.6.1.57
Phenylpyruvate
4.1.1.43
2.3.1.53
N-Acetyl-L-phenylalanine
1.1.1.222
1.1.1.237
Phenyllactate
1.13.11.27
2-Hydroxy-phenylacetate
1.14.13.-
2,6-Dihydroxy-phenylacetate
4.1.1.28
4.1.1.53
Phenylethylamine
1.4.3.4
1.4.3.6
1.4.99.4
Phenyl-ethylalcohol
1.1.1.90
Phenyl-acetaldehyde
1.2.1.39
1.2.1.5
3.5.1.4
Phenylacetate
4-Hydroxypnenylacetate
Tyrosine metabolism
3-Hydroxyphenylacetate
6.2.1.30
Phenyl-acetyl-CoA
2.3.1.-
2.3.1.14
Phenyl-acetylglycine
Phenyl-acetylglutamine
00360 3/22/08

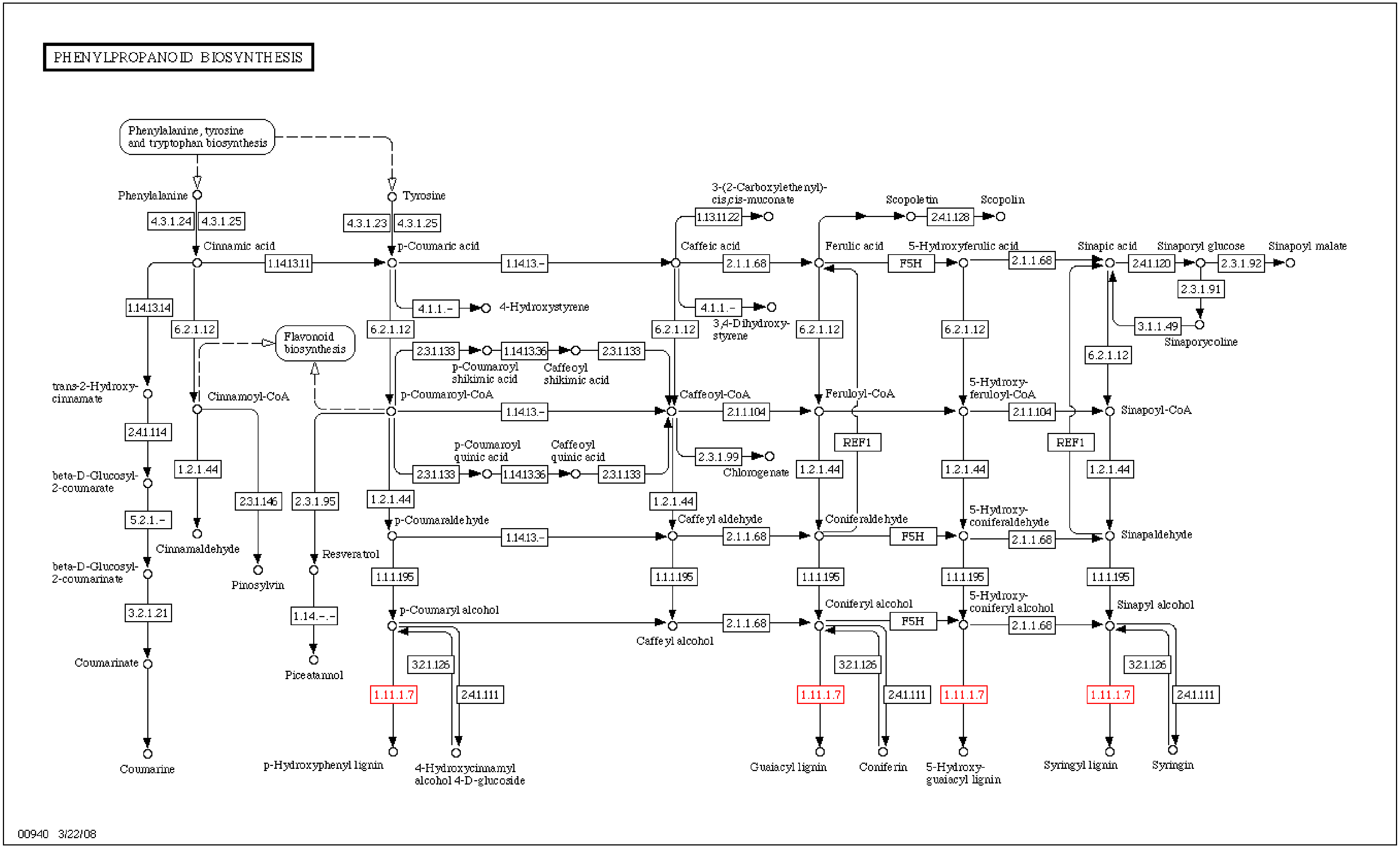

PHENYLPROPANOID BIOSYNTHESIS
Phenylalanine, tyrosine and tryptophan biosynthesis
Phenylalanine
Tyrosine
4.3.1.24
4.3.1.25
4.3.1.23
4.3.1.25
Cinnamic acid
1.14.13.11
p-Coumaric acid
1.14.13.-
Caffeic acid
2.1.1.68
Ferulic acid
F5H
5-Hydroxyferulic acid
Sinapic acid
2.4.1.120
Sinapoyl glucose
2.3.1.92
Sinapoyl malate
2.3.1.91
3.1.1.49
Sinapoylcholine
3-(2-Carboxyethenyl)-cis,cis-muconate
1.13.11.22
Scopoletin
2.4.1.128
Scopolin
4.1.1.-
4-Hydroxystyrene
3,4-Dihydroxystyrene
1.14.13.14
6.2.1.12
Flavonoid biosynthesis
2.3.1.133
1.14.13.36
p-Coumaroyl shikimic acid
Caffeoyl shikimic acid
trans-2-Hydroxycinnamate
Cinnamoyl-CoA
p-Coumaroyl-CoA
Caffeoyl-CoA
2.1.1.104
Feruloyl-CoA
5-Hydroxyferuloyl-CoA
Sinapoyl-CoA
REF1
2.4.1.114
p-Coumaroyl quinic acid
Caffeoyl quinic acid
2.3.1.99
Chlorogenate
beta-D-Glucosyl-2-coumarate
1.2.1.44
5.2.1.-
2.3.1.146
2.3.1.95
Cinnamaldehyde
Pinosylvin
Resveratrol
p-Coumaraldehyde
Caffeyl aldehyde
Coniferaldehyde
5-Hydroxyconiferaldehyde
Sinapaldehyde
beta-D-Glucosyl-2-coumarinate
1.1.1.195
1.14.-.-
3.2.1.21
p-Coumaryl alcohol
Caffeyl alcohol
Coniferyl alcohol
5-Hydroxyconiferyl alcohol
Sinapyl alcohol
Piceatannol
Coumarinate
3.2.1.126
2.4.1.111
1.11.1.7
Coumarine
p-Hydroxyphenyl lignin
4-Hydroxycinnamyl alcohol 4-D-glucoside
Guaiacyl lignin
Coniferin
5-Hydroxyguaiacyl lignin
Syringyl lignin
Syringin
00940 3/22/08

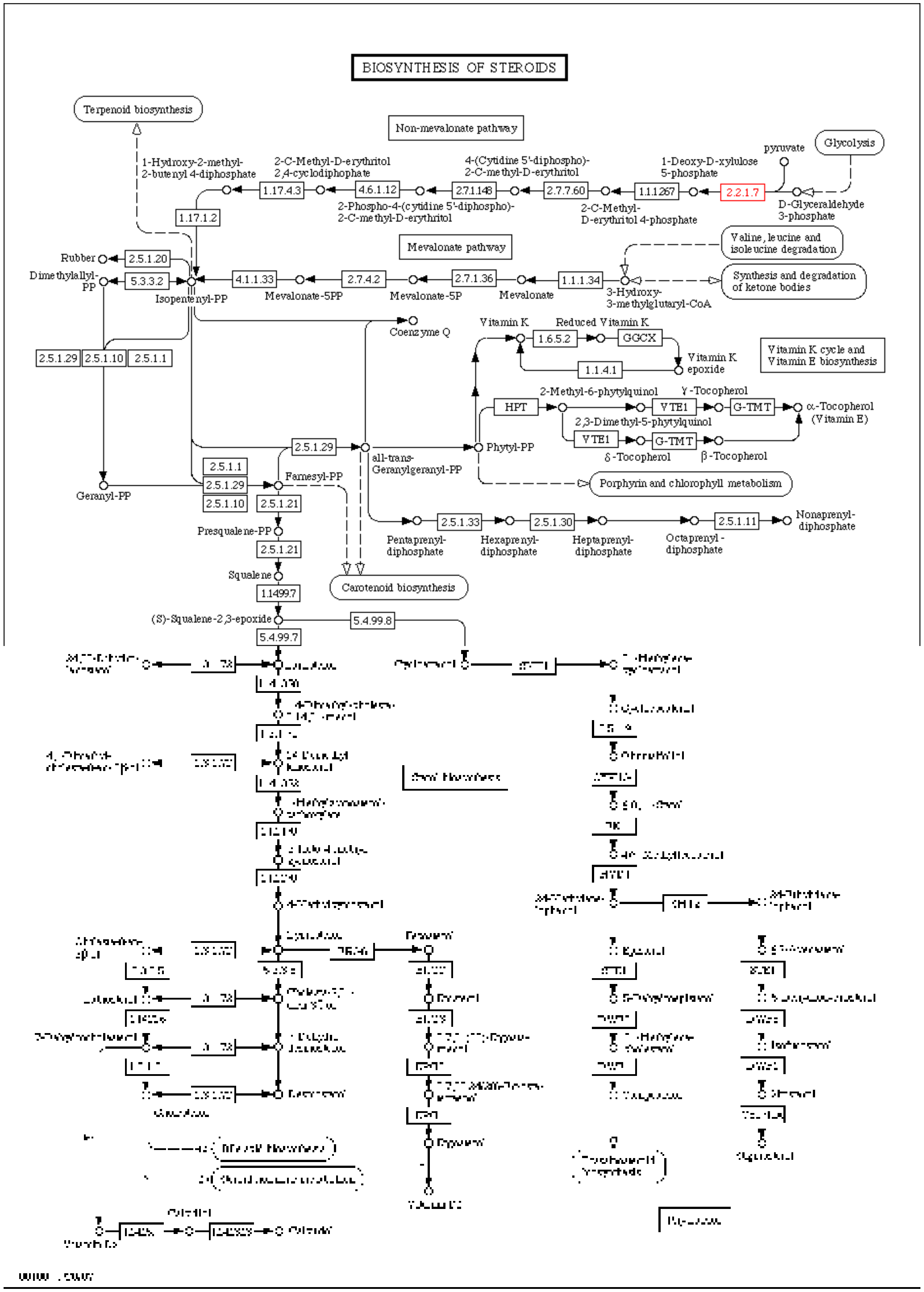
BIOSYNTHESIS OF STEROIDS
Terpenoid biosynthesis
Non-mevalonate pathway
Glycolysis
pyruvate
1-Hydroxy-2-methyl-2-butenyl 4-diphosphate
2-C-Methyl-D-erythritol 2,4-cyclodiphosphate
4-(Cytidine 5'-diphospho)-2-C-methyl-D-erythritol
1-Deoxy-D-xylulose 5-phosphate
1.17.4.3
4.6.1.12
2.7.1.148
2.7.7.60
1.1.1.267
2.2.1.7
D-Glyceraldehyde 3-phosphate
2-Phospho-4-(cytidine 5'-diphospho)-2-C-methyl-D-erythritol
2-C-Methyl-D-erythritol 4-phosphate
1.17.1.2
Mevalonate pathway
Valine, leucine and isoleucine degradation
Synthesis and degradation of ketone bodies
Rubber
2.5.1.20
Dimethylallyl-PP
5.3.3.2
4.1.1.33
2.7.4.2
2.7.1.36
1.1.1.34
Isopentenyl-PP
Mevalonate-5PP
Mevalonate-5P
Mevalonate
3-Hydroxy-3-methylglutaryl-CoA
Coenzyme Q
Vitamin K
Reduced Vitamin K
1.6.5.2
GGCX
Vitamin K epoxide
1.1.4.1
Vitamin K cycle and Vitamin E biosynthesis
2.5.1.29
2.5.1.10
2.5.1.1
2-Methyl-6-phytylquinol
γ-Tocopherol
HPT
VTE1
G-TMT
α-Tocopherol (Vitamin E)
2,3-Dimethyl-5-phytylquinol
δ-Tocopherol
β-Tocopherol
all-trans-Geranylgeranyl-PP
Phytyl-PP
Porphyrin and chlorophyll metabolism
Geranyl-PP
Farnesyl-PP
2.5.1.21
2.5.1.33
2.5.1.30
2.5.1.11
Nonaprenyl-diphosphate
Pentaprenyl-diphosphate
Hexaprenyl-diphosphate
Heptaprenyl-diphosphate
Octaprenyl-diphosphate
Presqualene-PP
Squalene
1.14.99.7
Carotenoid biosynthesis
(S)-Squalene-2,3-epoxide
5.4.99.8
5.4.99.7

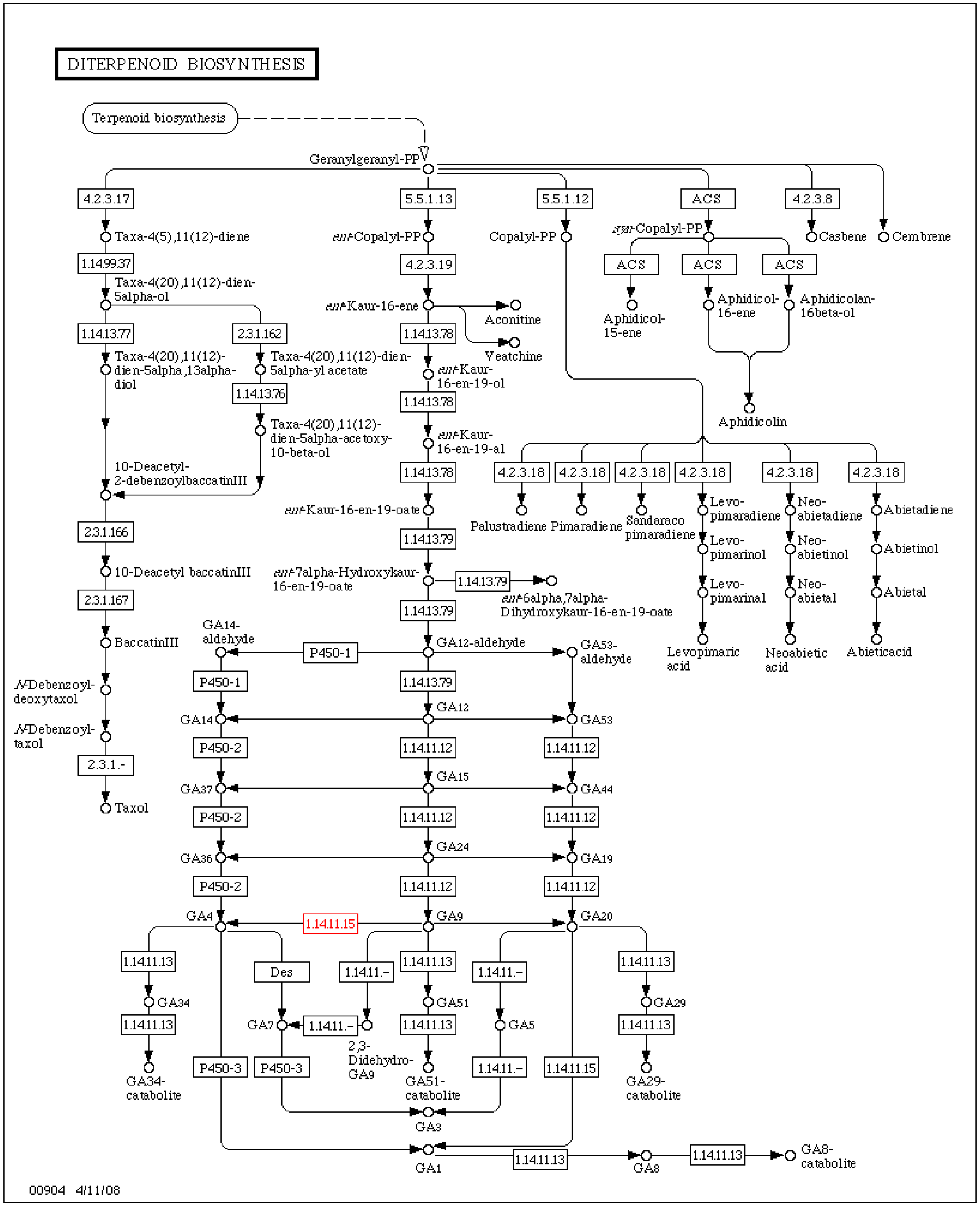
DITERPENOID BIOSYNTHESIS
Terpenoid biosynthesis
Geranylgeranyl-PP
4.2.3.17
5.5.1.13
5.5.1.12
ACS
4.2.3.8
Taxa-4(5),11(12)-diene
ent-Copalyl-PP
Copalyl-PP
syn-Copalyl-PP
Casbene
Cembrene
1.14.99.37
4.2.3.19
ACS
ACS
ACS
Taxa-4(20),11(12)-dien-5alpha-ol
ent-Kaur-16-ene
Aconitine
Aphidicol-15-ene
Aphidicol-16-ene
Aphidicolan-16beta-ol
1.14.13.77
2.3.1.162
1.14.13.78
Veatchine
Taxa-4(20),11(12)-dien-5alpha,13alpha-diol
Taxa-4(20),11(12)-dien-5alpha-yl acetate
ent-Kaur-16-en-19-ol
1.14.13.76
1.14.13.78
Aphidicolin
Taxa-4(20),11(12)-dien-5alpha-acetoxy-10-beta-ol
ent-Kaur-16-en-19-al
10-Deacetyl-2-debenzoylbaccatinIII
1.14.13.78
4.2.3.18
4.2.3.18
4.2.3.18
4.2.3.18
4.2.3.18
4.2.3.18
ent-Kaur-16-en-19-oate
Palustradiene
Pimaradiene
Sandaracopimaradiene
Levo-pimaradiene
Neo-abietadiene
Abietadiene
2.3.1.166
1.14.13.79
Levo-pimarinol
Neo-abietinol
Abietinol
10-Deacetyl baccatinIII
ent-7alpha-Hydroxykaur-16-en-19-oate
1.14.13.79
ent-6alpha,7alpha-Dihydroxykaur-16-en-19-oate
Levo-pimarinal
Neo-abietal
Abietal
2.3.1.167
1.14.13.79
BaccatinIII
GA14-aldehyde
P450-1
GA12-aldehyde
GA53-aldehyde
Levopimaric acid
Neoabietic acid
Abieticacid
N-Debenzoyl-deoxytaxol
P450-1
1.14.13.79
GA14
GA12
GA53
N-Debenzoyl-taxol
P450-2
1.14.11.12
1.14.11.12
2.3.1.-
GA37
GA15
GA44
Taxol
P450-2
1.14.11.12
1.14.11.12
GA36
GA24
GA19
P450-2
1.14.11.12
1.14.11.12
GA4
1.14.11.15
GA9
GA20
1.14.11.13
Des
1.14.11.-
1.14.11.13
1.14.11.-
1.14.11.13
GA34
GA51
GA29
1.14.11.13
GA7
1.14.11.-
2,3-Didehydro-GA9
1.14.11.13
GA5
1.14.11.13
P450-3
P450-3
1.14.11.-
1.14.11.15
GA34-catabolite
GA51-catabolite
GA29-catabolite
GA3
GA1
1.14.11.13
GA8
1.14.11.13
GA8-catabolite
00904 4/11/08

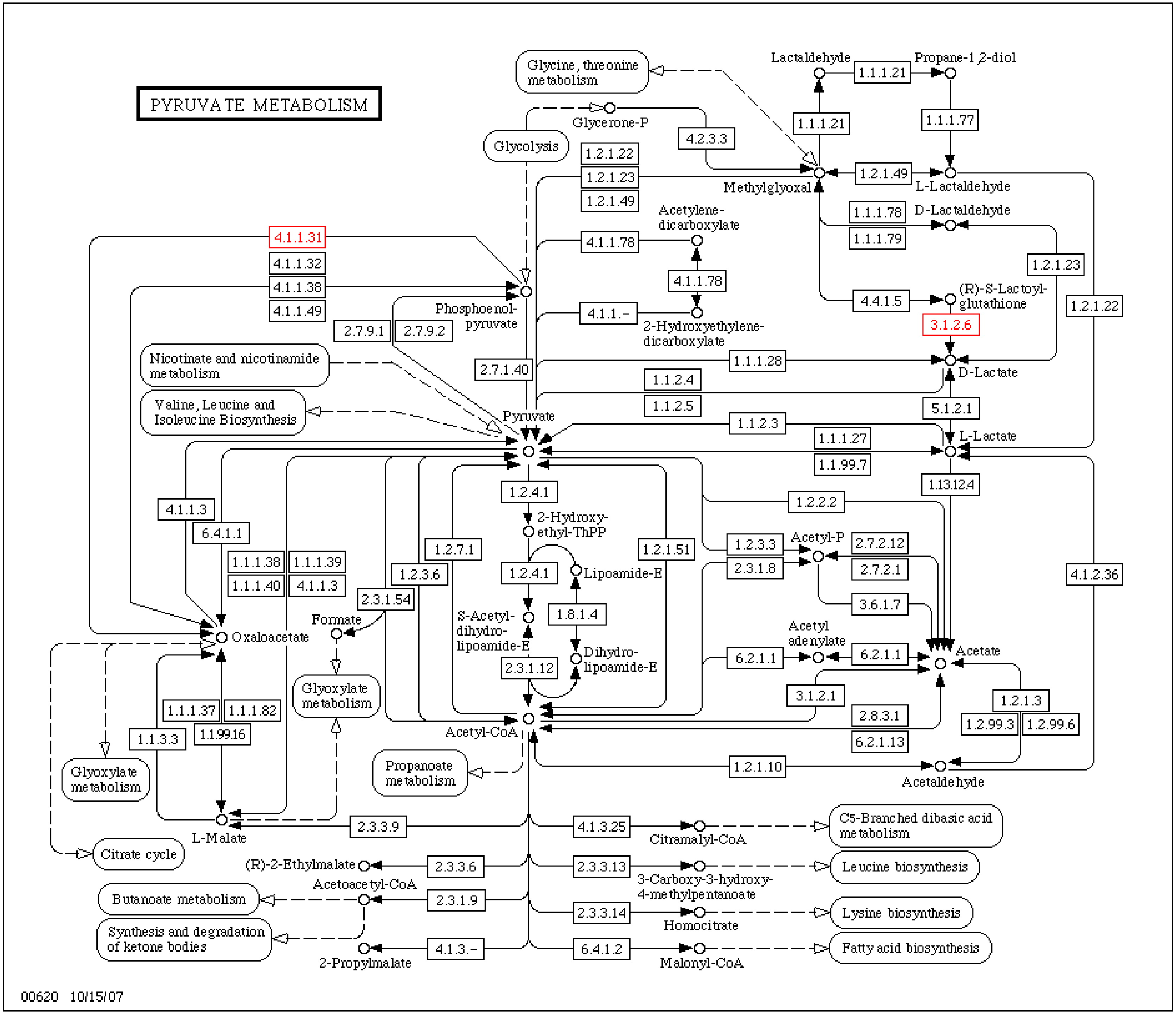

PYRUVATE METABOLISM
Glycine, threonine metabolism
Glycerone-P
Glycolysis
4.2.3.3
1.2.1.22
1.2.1.23
1.2.1.49
Lactaldehyde
1.1.1.21
Propane-1,2-diol
1.1.1.21
1.1.1.77
1.2.1.49
Methylglyoxal
L-Lactaldehyde
1.1.1.78
1.1.1.79
D-Lactaldehyde
Acetylene-dicarboxylate
4.1.1.78
4.1.1.78
4.1.1.-
2-Hydroxyethylene-dicarboxylate
1.2.1.23
4.4.1.5
(R)-S-Lactoyl-glutathione
3.1.2.6
1.2.1.22
4.1.1.31
4.1.1.32
4.1.1.38
4.1.1.49
Phosphoenol-pyruvate
2.7.9.1
2.7.9.2
2.7.1.40
Nicotinate and nicotinamide metabolism
Valine, Leucine and Isoleucine Biosynthesis
1.1.1.28
D-Lactate
1.1.2.4
1.1.2.5
5.1.2.1
Pyruvate
1.1.2.3
L-Lactate
1.1.1.27
1.1.99.7
1.13.12.4
4.1.1.3
6.4.1.1
1.2.4.1
2-Hydroxy-ethyl-ThPP
1.2.2.2
1.1.1.38
1.1.1.39
1.1.1.40
4.1.1.3
1.2.7.1
1.2.3.6
1.2.1.51
1.2.3.3
Acetyl-P
2.7.2.12
2.3.1.8
2.7.2.1
3.6.1.7
4.1.2.36
1.2.4.1
Lipoamide-E
2.3.1.54
Formate
S-Acetyl-dihydro-lipoamide-E
1.8.1.4
Oxaloacetate
Dihydro-lipoamide-E
Acetyl adenylate
6.2.1.1
6.2.1.1
Acetate
2.3.1.12
3.1.2.1
1.2.1.3
Glyoxylate metabolism
2.8.3.1
1.2.99.3
1.2.99.6
1.1.1.37
1.1.1.82
Acetyl-CoA
1.1.3.3
1.1.99.16
6.2.1.13
Glyoxylate metabolism
Propanoate metabolism
1.2.1.10
Acetaldehyde
L-Malate
2.3.3.9
4.1.3.25
Citramalyl-CoA
C5-Branched dibasic acid metabolism
Citrate cycle
(R)-2-Ethylmalate
2.3.3.6
2.3.3.13
Leucine biosynthesis
3-Carboxy-3-hydroxy-4-methylpentanoate
Acetoacetyl-CoA
Butanoate metabolism
2.3.1.9
2.3.3.14
Homocitrate
Lysine biosynthesis
Synthesis and degradation of ketone bodies
4.1.3.-
6.4.1.2
Fatty acid biosynthesis
2-Propylmalate
Malonyl-CoA
00620 10/15/07

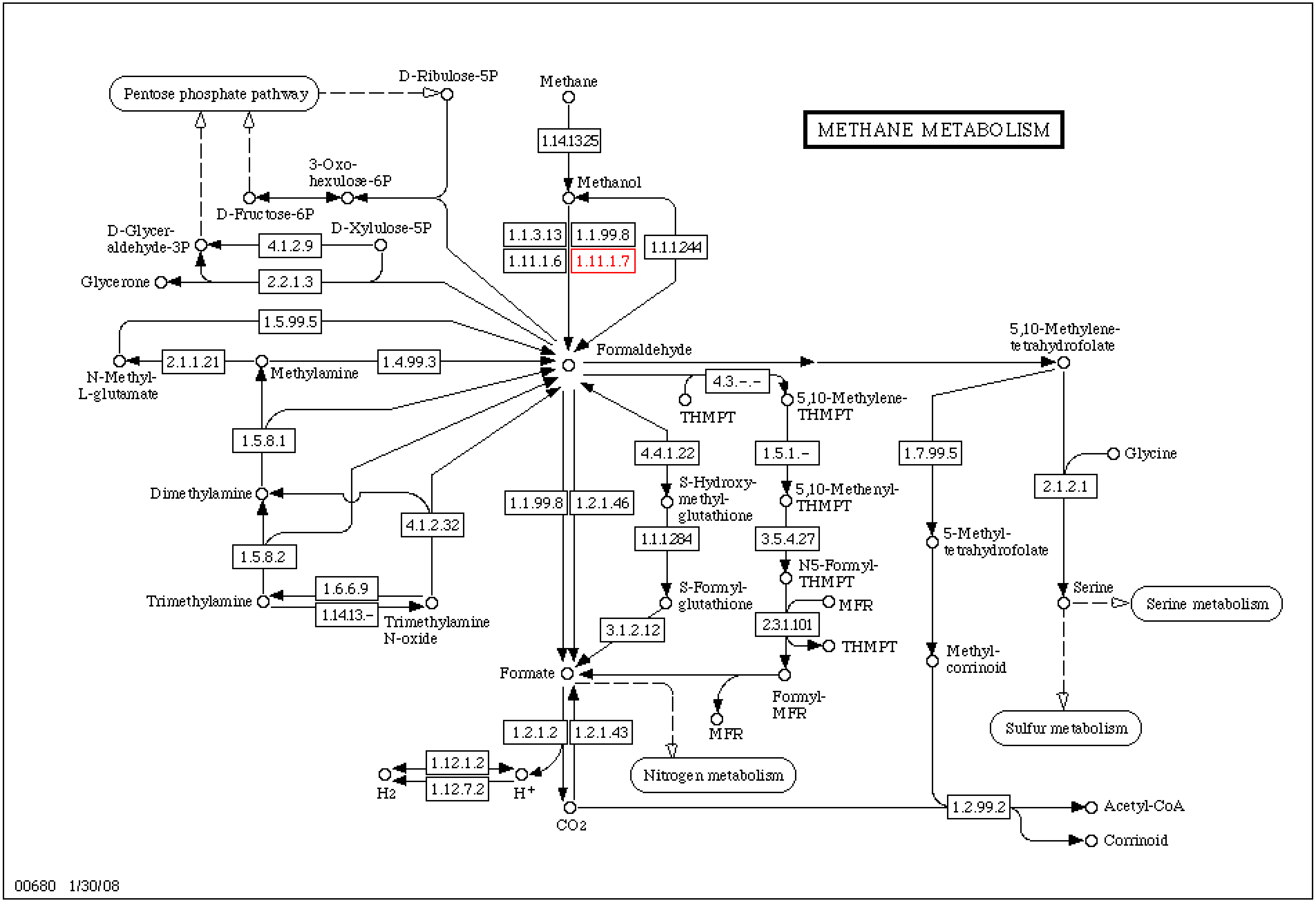
METHANE METABOLISM
Pentose phosphate pathway
D-Ribulose-5P
Methane
1.14.13.25
Methanol
3-Oxo-hexulose-6P
D-Fructose-6P
D-Glyceraldehyde-3P
4.1.2.9
D-Xylulose-5P
Glycerone
2.2.1.3
1.1.3.13
1.1.99.8
1.11.1.6
1.11.1.7
1.1.1.244
1.5.99.5
2.1.1.21
Methylamine
1.4.99.3
Formaldehyde
N-Methyl-L-glutamate
5,10-Methylene-tetrahydrofolate
4.3.-.-
THMPT
5,10-Methylene-THMPT
1.5.8.1
4.4.1.22
1.5.1.-
1.7.99.5
Glycine
2.1.2.1
Dimethylamine
S-Hydroxymethylglutathione
5,10-Methenyl-THMPT
1.1.99.8
1.2.1.46
4.1.2.32
1.1.1.284
3.5.4.27
5-Methyl-tetrahydrofolate
1.5.8.2
N5-Formyl-THMPT
1.6.6.9
Trimethylamine
S-Formylglutathione
MFR
Serine
Serine metabolism
1.14.13.-
Trimethylamine N-oxide
3.1.2.12
2.3.1.101
THMPT
Methylcorrinoid
Formate
Formyl-MFR
MFR
1.2.1.2
1.2.1.43
Sulfur metabolism
1.12.1.2
1.12.7.2
Nitrogen metabolism
H2
H+
CO2
1.2.99.2
Acetyl-CoA
Corrinoid
00680 1/30/08
